# Powerful QTL mapping and favorable allele mining in an all-in-one population: a case study of heading date

**DOI:** 10.1093/nsr/nwae222

**Published:** 2024-06-26

**Authors:** Pengfei Wang, Ying Yang, Daoyang Li, Zhichao Yu, Bo zhang, Xiangchun Zhou, Lizhong Xiong, Jianwei Zhang, Lei Wang, Yongzhong Xing

**Affiliations:** National Key Laboratory of Crop Genetic Improvement, Hubei Hongshan Laboratory, Huazhong Agricultural University, Wuhan 430070, China; Institute of Tropical Crop Genetic Resources, Chinese Academy of Tropical Agricultural Sciences, Haikou 571101, China; National Key Laboratory of Crop Genetic Improvement, Hubei Hongshan Laboratory, Huazhong Agricultural University, Wuhan 430070, China; National Key Laboratory of Crop Genetic Improvement, Hubei Hongshan Laboratory, Huazhong Agricultural University, Wuhan 430070, China; National Key Laboratory of Crop Genetic Improvement, Hubei Hongshan Laboratory, Huazhong Agricultural University, Wuhan 430070, China; National Key Laboratory of Crop Genetic Improvement, Hubei Hongshan Laboratory, Huazhong Agricultural University, Wuhan 430070, China; National Key Laboratory of Crop Genetic Improvement, Hubei Hongshan Laboratory, Huazhong Agricultural University, Wuhan 430070, China; National Key Laboratory of Crop Genetic Improvement, Hubei Hongshan Laboratory, Huazhong Agricultural University, Wuhan 430070, China; National Key Laboratory of Crop Genetic Improvement, Hubei Hongshan Laboratory, Huazhong Agricultural University, Wuhan 430070, China; National Key Laboratory of Crop Genetic Improvement, Hubei Hongshan Laboratory, Huazhong Agricultural University, Wuhan 430070, China; National Key Laboratory of Crop Genetic Improvement, Hubei Hongshan Laboratory, Huazhong Agricultural University, Wuhan 430070, China; Yazhouwan National Laboratory, Sanya 572024, China

**Keywords:** MAGIC population, population structure, bin map, novel casual variant, superior allele

## Abstract

The multiparent advanced generation intercross (MAGIC) population is characterized with great potentials in power and resolution of quantitative trait locus (QTL) mapping, but single nucleotide polymorphism (SNP)-based GWAS does not fully reach its potential. In this study, a MAGIC population of 1021 lines was developed from four Xian and four Geng varieties from five subgroups of rice. A total of 44 000 genes showed functional polymorphisms among eight parents, including frameshift variations or premature stop codon variations, which provides the potential to map almost all genes of the MAGIC population. Principal component analysis results showed that the MAGIC population had a weak population structure. A high-density bin map of 24 414 bins was constructed. Segregation distortion occurred in the regions possessing the genes underlying genetic incompatibility and gamete development. SNP-based association analysis and bin-based linkage analysis identified 25 significant loci and 47 QTLs for heading date, including 14 known heading date genes. The mapping resolution of genes is dependent on genetic effects with offset distances of <55 kb for major effect genes and <123 kb for moderate effect genes. Four causal variants and noncoding structure variants were identified to be associated with heading date. Three to four types of alleles with strong, intermediate, weak, and no genetic effects were identified from eight parents, providing flexibility for the improvement of rice heading date. In most cases, japonica rice carries weak alleles, and indica rice carries strong alleles and nonfunctional alleles. These results confirm that the MAGIC population provides the exceptional opportunity to detect QTLs, and its use is encouraged for mapping genes and mining favorable alleles for breeding.

## INTRODUCTION

There are 27 species in the genus *Oryza*, which are widely distributed in tropical and subtropical regions [1]. *Oryza sativa* is the most important staple food in Asia and a model species for scientific crop research. There are two main *O. sativa* subspecies, indica/Xian and japonica/Geng. The estimation of the population-differentiation statistic (F_ST_) between the indica and japonica landraces yielded a value of 0.55, signifying a substantial degree of population differentiation [2]. The genetic diversity of Geng rice is much lower than that of Xian rice [3]. Geng rice predominantly occupies high latitudes and altitudes, such as Northeast China and Yunnan Province. In contrast, Xian rice exhibits a more extensive distribution area across tropical and subtropical regions, encompassing a broader range of climatic conditions. Due to reproductive isolation, gene flow between Xian and Geng is very limited, which prevents the development of primary intersubspecies populations for QTL mapping and hybrid breeding [4]. Geng rice originated from wild *Oryza rufipogon* in southern China [5]. Xian rice originated from a hybridization event between *O. rufipogon* and *Oryza nivara*, leading to the current geographical distribution pattern and genetic characteristics observed in *Oryza sativa* [6].

One notable advantage of plant genetics research is the readily accessible experimental population. By employing diverse genetic designs, the genomes of distinct founders are intermixed, and genetic recombination is allocated to various progeny, facilitating investigations into the intricate interplay between genes, traits and the environment. Conventional approaches for genetic mapping have traditionally utilized biparental populations such as F_2_, recombinant inbred lines (RILs), and introgression lines [7]. In these biparental populations, only two alleles are separated at any polymorphic locus, thereby hindering the simultaneous comparison of genetic effects among multiple alleles in rice germplasm. Association mapping frequently employs extensive germplasm collections encompassing a wide array of genetic diversity to elucidate the connections between phenotypes and genotypes via historical linkage disequilibrium. Such germplasm populations have played an important role in genetic mapping of major crops over the past two decades [2,8,9]. However, the presence of population structure within the germplasm population represents a pivotal genetic factor that contributes to spurious associations. Even though some advanced statistical methods have been proposed to control noise from genetic structure and kinship, spurious QTLs are still a problem in association studies [10]. Additionally, the prevalence of novel functional variations tends to exhibit low-frequency occurrences [11]. Thus, these novel functional alleles often fail to be identified because they are ignored in association analyses [12]. The construction of multiparent advanced generation intercross (MAGIC) populations primarily involves hybridizing multiple parental genomes to attain a diverse genetic foundation. Subsequently, meticulous preservation of genetic diversity is achieved through single-seed descent, ensuring that each progeny incorporates genetic contributions from all parents [13]. Multiple rounds of extensive recombination across hybrid generations effectively disrupt population structure, facilitating more robust analyses. The utilization of MAGIC populations has been advocated to enhance the power and resolution of QTL mapping. However, the untapped potential of the MAGIC population is evident with two prominent challenges concerning the precision of genotyping and the efficiency of genetic statistical detection [14].

The advancement of sequencing technology has increased the feasibility of conducting large-scale high-density genotyping [8]. In some studies, GWAS at the single nucleotide polymorphism (SNP) level has been conducted with MAGIC populations [15,16], in which the pedigree information was not utilized for QTL mapping. Nevertheless, resequencing-based genotyping is not entirely devoid of errors. To address this issue, a strategy involving the conversion of fully linked SNPs within the genome of the offspring population into bin genotypes has been devised [17,18]. This approach aims to rectify the genotyping inaccuracies associated with high-throughput sequencing. Bin-level analysis has already exhibited advantages in QTL mining with a MAGIC population. For example, in rice, three previously reported heading date genes were successfully identified, and the genetic effects of parental alleles were estimated at the bin level in a four-way MAGIC population encompassing 247 RILs [19]. Similarly, in maize, a CUBIC (complete-diallel design plus unbalanced breeding-like intercross) population consisting of 1404 RILs derived from 24 inbred lines facilitated the discovery of numerous QTLs associated with 23 agronomic traits at the SNP and bin levels [20]. In addition to rice and maize, GWAS at bin-level have been performed in the MAGIC population of cotton [21]. While the current model for GWAS at bin level does not utilize the linkage information which is able to be tracked.

Here, an eight-way MAGIC population was developed by crossing four Xian and four Geng cultivars to confirm the strong power and high resolution of QTL mapping and rank the genetic effects of eight parental alleles. The main aims of this study were to: (1) compare the power and resolution of QTL mapping via GWAS between a germplasm collection and a MAGIC population, (2) perform linkage analysis by constructing a high-density bin map and (3) further mine favorable alleles for breeding.

## RESULTS

### Numerous functionally differentiated genes among the eight founders

**Figure 1.**
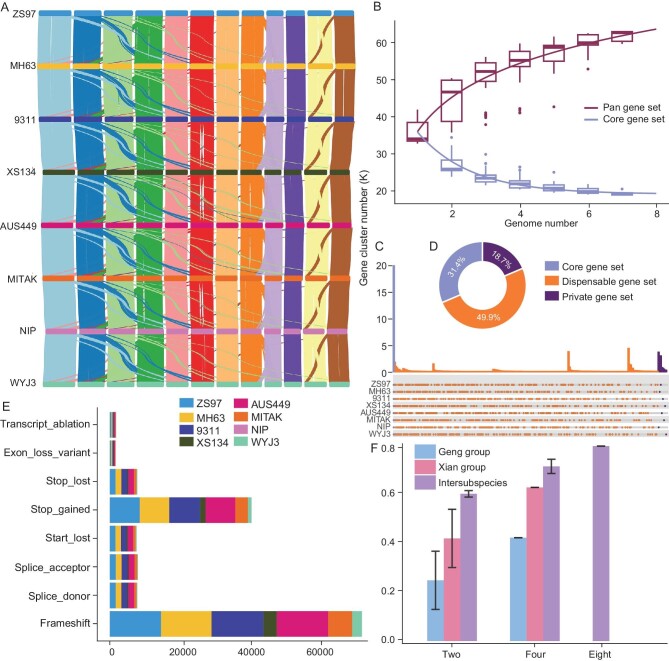
Gene function differences among the eight founders. A. Genome collinearity of eight parents based on homologous genes. The color coding of the eight parental individuals remains consistent across all subsequent images. B. Pan gene set and core gene set simulated by gene cluster number. C. The number of core gene sets, different dispensable gene sets and private gene sets. D. The proportions of the core gene set, dispensable gene set and private gene set. E. The number of nucleotide variations with a high probability of affecting gene function in the seven parents. F. The rate of differential alleles in biparental, four-way and eight-way different intrasubspecies and intersubspecies cross combinations among the eight parents.

The reference genomes of four founders, ZS97, MH63 [22], 9311 [23] and NIP [24], were available in public databases. For the remaining four founders XS134, AUS449, MITAK and WYJ3, we generated high-fidelity reads ranging from 16.8 to 37.5 Gb in size, with a read length N50 exceeding 13.5 kb (Table S1). The four newly assembled genomes ranged from 380–385 Mb, with a Hifiasm contig size of 27.4–30.2 Mb. The whole genome was evaluated, the BUSCO score was 98.6%–98.9%, and the LAI score was 21.29–23.95 (Table S2). The dot plots and synteny plots generated from genome comparisons among the eight founders consistently exhibited strong collinearity, indicating a high degree of alignment between their respective genomes (Fig. S1). These evaluations indicated high continuity and high completeness of the four founders. A total of 59 148 to 59 776 genes were annotated in single genomes, of which 40 149 to 40 683 genes were protein-coding genes (Table S3). BUSCO analysis based on the protein-coding genes showed that the accuracy of the annotations was notably high, ranging from 94.3% to 95.2%. This finding underscores the high quality of gene annotation achieved. Additionally, the gene alignment block demonstrated robust collinearity, further supporting the reliability and consistency of the genomic annotations (Fig. 1A).

Utilizing the gene annotations from the eight genomes, we assembled a comprehensive pan gene set. This pan gene set encompassed a total of 379 216 genes, categorized into 65 236 distinct gene subsets. The subsets included the core gene set present in all eight genomes, comprising 20 488 gene sets. Additionally, the dispensable gene set comprised genes present in 2 to 7 genomes, amounting to 32 591 gene sets. Furthermore, we identified a private gene set consisting of 12 157 unique gene sets found in a single genome (Fig. 1B–D). Taking Nipponbare as the reference genome, DNA variants were identified between the remaining seven parents and Nipponbare. The observed variations included 273 220 to 2 592 459 SNPs, 68 293 to 483 557 insertions and deletions (InDels), 4209 to 17 865 structural variations (SVs) larger than 50 bases, and 60 to 252 inversions. There were 4 690 550 SNPs, 990 659 InDels and 42 389 SVs among the eight parents. A subset of these SVs, ranging from 2136 to 8886, was found within the upstream and downstream regulatory regions of genes. Furthermore, 339 to 1790 SVs were identified within exons, while 140 to 963 SVs were located within the untranslated region (UTR) of genes (Fig. S2). Among these variants, we identified a range of 2845 to 15 149 genes with frameshift variants. Additionally, a subset of genes, from 1028 to 8999, exhibited premature termination variants, indicating potential disruptions in the protein-coding sequences of these genes (Fig. 1E). That is, regardless of the variations in the noncoding region, at least 44 000 genes (79.8%) exhibited functional and nonfunctional polymorphisms in the eight parents, which could potentially be mapped in the MAGIC population if the corresponding phenotypes were precisely collected (Fig. 1F).

### Weak population structure in the MAGIC population

**Figure 2.**
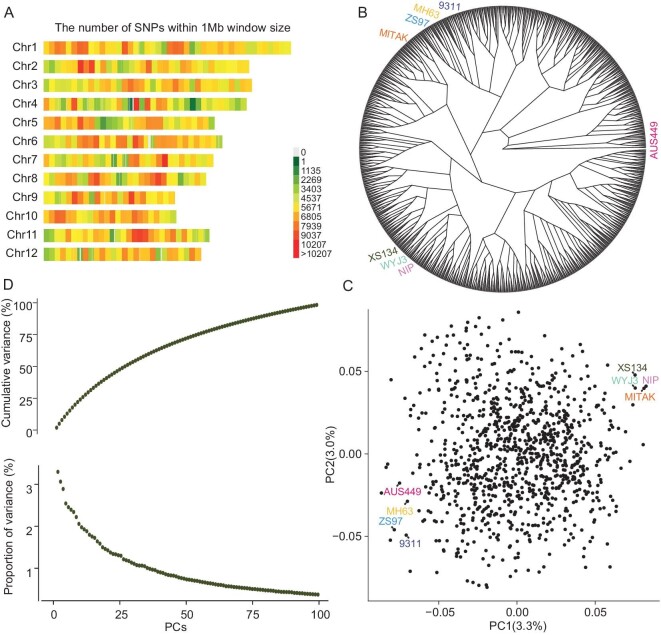
Population structure of the MAGIC population. A. Distribution of SNPs in 12 chromosomes. B. Neighbor-joining tree of the eight founders and 1021 MAGIC RILs. C. PCA plot of eight founders and 1021 MAGIC RILs. D. Variance of the first 100 PCs by principal component analysis. The x-axis represents the descending order of the first 100 principal components by variance explaining, and the y-axis represents cumulative variance (%) and proportion of variance contributed by single principal components, respectively.

More than 5 Gb of high-quality resequencing data were generated for each of 1021 MAGIC lines (Table S4). Following stringent quality control, a total of 2 184 595 high-quality SNP variants were obtained. The distribution of these SNPs varied across different chromosomes, with the highest density of 6.6 SNPs/kb on chromosome 10 and the lowest density of 4.8 SNPs/kb on chromosome 4 (Fig. 2A). Approximately 2.6% of SNP genotypes were heterozygous across the population, which slightly exceeded the expected frequency after six generations of self-pollination (1/2^6^) (Table S5).

A set of 63 748 evenly distributed SNPs was subsequently used to construct a neighbor-joining phylogenetic tree. No distinct population structure was identified in the MAGIC population, and the four offspring from the same eight-way F_1_ plant tended to cluster together (Fig. 2B) (Data 1). Principal component analysis (PCA) results showed that the first two principal components (PCs) accounted for only 6.3% of the total variance in the MAGIC population, suggesting the achievement of full recombination among eight parents and the absence of discernible population stratification (Fig. 2C). This observation is further supported by the cumulative curve depicting the variance explained by each PC (Fig. 2D). LD decay extended to a distance from 611 to 2141 kb across chromosomes (r^2^ = 0.2). Notably, numerous LD regions were observed throughout the genome, particularly in centromeric regions on chromosomes 1, 2 and 5, where high LD was evident. The centromeric regions exhibited low LD on chromosomes 8 and 10 (Fig. S3).

### High-density genome-wide bin map of the MAGIC population

**Figure 3.**
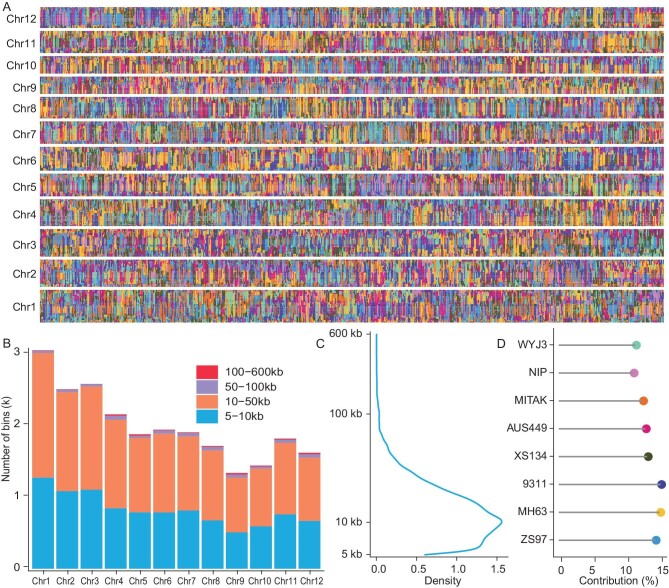
Whole-genome bin construct in the MAGIC population. A. The bin map of the MAGIC population; each column represents an RIL. B. Distribution of bin lengths across 12 chromosomes at the population level. C. Density plot of bin lengths at the population level. D. Contribution of the eight founders at the population level.

To gain deeper insights into the population composition, hidden Markov model (HMM)-based ancestry inference was employed to transfer the biallelic SNP genotypes to the corresponding parental bins. A total of 24 144 bins were identified in the 1021 MAGIC lines (Fig. 3A). The bin lengths ranged from 5 kb to 591.5 kb, with an average length of 15.5 kb (Fig. 3C). Most bins (97.3%) were smaller than 50 kb, and 41.3% of the bins were smaller than 10 kb (Fig. 3B). The longest bin was located on chromosome 6 (position 18 220 256–18 811 707), where a well-known ∼4.3 Mb inversion variant associated with the differentiation between Xian and Geng rice has been reported [25]. Our comprehensive genome and gene synteny analysis results corroborated the existence of this inversion (Fig. 1A; Figs S2B, S4B). The number of bins varied from 66 to 597 across individual lines, with an average of 128.3 bins per line. For instance, line MG4 exhibited a total of 74 bins, with the longest bin spanning 21.6 Mb on chromosome 2 and the shortest bin spanning 92.8 kb on chromosome 1 (Fig. S4A). Two hundred MAGIC lines were randomly sampled 100 times to calculate the average number of bins at the population level. The permutation test showed that the average number was 11 679, which was 7.8 times greater than that in the biparental population and 2.2 times greater than that in the four-way MAGIC population with a similar population size [26,27]. Therefore, the eight-way MAGIC population has the potential to enhance mapping resolution.

### Segregation distortion prefers occurrence in the region harboring incompatibility loci

It is expected that each founder equally contributed to the population because we performed pairwise crossing until we obtained eight-way F_1_ hybrids. Segregation distortion occurred throughout the genome (Fig. S5A). In general, parents XS134 and AUS449 demonstrated adherence to a ratio of one-eighth in the population, and the genetic contribution of Xian rice parents, ZS97, MH63 and 9311, significantly outweighed that of the Geng rice parents, MITAK, NIP and WYJ3. The percentage of Xian accounted for 54.4%, surpassing that of Geng (Fig. 3D). A comprehensive analysis identified a total of 119 segregation distortion regions distributed across the entire genome, encompassing a cumulative length of 304.6 Mb (Table S6). This indicates that nearly 80% of the genome regions exhibited segregation distortion.

Thirteen regions exhibited the strongest segregation distortion. They harbored genetic incompatibility loci, including the well-characterized *Sa, Sc, S5, S7, pf12, HSA1, DPL1* and *DPL2* (Fig. S5B). The alleles of three Xian rice varieties, ZS97, MH63 and 9311, had a significantly higher frequency than the alleles of three Geng rice varieties, XS134, NIP and WYJ3, in the region surrounding *S5*, the major locus associated with Xian-Geng genetic incompatibility [28]. Sequence analysis revealed that ZS97, MH63 and 9311 carry functional *ORF3* and *ORF5*, while XS134, NIP and WYJ3 carry functional *ORF4*. In the heterozygous state, the presence of functional *ORF5* in combination with *ORF4* leads to the elimination of female gametes in the absence of *ORF3* protection. This suggests that an increase in the proportion of individuals carrying both functional *ORF3* and *ORF5* is expected, and the observed distribution aligns precisely with the predicted segregation pattern. *Pf12*, a key determinant of pollen fertility in Xian-Geng genetic incompatibility [29,30], exhibited a higher frequency in the four parent lines ZS97, MH63, 9311 and XS134 than in AUS449, MITAK, NIP and WYJ3. Sequence analysis of these eight parental alleles confirmed that the functional allele *pf12a* was present in ZS97, MH63, 9311 and XS134, a frameshift variant of 1-bp deletion in exon 1 was detected in AUS449 causing a non-functional *pf12a* allele, and *pf12a* was completely absent in MITAK, NIP and WYJ3 (Table S7). The male gametes carrying nonfunctional *pf12a* were specifically killed in heterozygous conditions [29,30], which well explained the decreased allele frequency of nonfunctional *pf12a* alleles in the MAGIC population (Fig. S6).

In addition, 18 segregation distortion regions were colocalized with the genes associated with male and female gamete development as well as heading (Fig. S5C). Moreover, the eight founders possessed functional variations at these loci (Table S8). *Hd1, Ghd7* and *Ghd8* are photoperiod flowering genes. The functional allele combination *Ghd7Ghd8Hd1* results in a very delayed heading in Wuhan, which leads to unfilled grains [31]. It is expected that functional alleles of *Hd1, Ghd7* or *Ghd8* would be selected in the process of developing the MAGIC population. ZS97, XS134, NIP and WYJ3 possessed the functional *Hd1* allele. Interestingly, the functional *Hd1* allele frequencies of ZS97, XS134, NIP and WYJ3 were indeed reduced to 39.1%, and the observed frequency of the functional allele combination of *Ghd7Ghd8Hd1* was 1.5%, which was less than the expected frequency of 3.1%.

### Whole-genome recombination hotspots and recombination suppression

A genetic linkage map covering 1893.9 cM was developed with bins in the MAGIC population (Fig. S7). The genetic neighboring markers <200 kb on chromosomes 4, 6, 10 and 12 covered 5.9 cM to 11.7 cM, which suggested recombination hotspots in these very small regions (Fig. S7A). To identify recombination hotspots and suppression, we calculated the number of bins per Mb and recombination rate per Mb in the MAGIC population. The number of bins per Mb varied from 10 to 89 (Fig. S8). The centromere region of chromosome 5 showed the most pronounced recombination suppression, with the smallest recombination of 10 bins per Mb. There was a significant positive correlation between the number of bins per Mb and the recombination rate per Mb (R = 0.49, *P* < 2e-16) (Fig. S9A). A total of 1206 breakpoints were characterized as recombination hotspots, and 1212 breakpoints were characterized as recombination suppression (Table S9). These hotspot regions exhibited significantly higher levels of gene expression across the four tissues than the surrounding background and suppression regions (Fig. S9B). Additionally, the chromatin accessibility, histone modification and DNA methylation at these recombination hotspots were markedly distinct from those observed in the background regions and recombination suppression (Fig. S9C).

Inversions have been recognized as key factors influencing genetic recombination [32]. The inversion regions displayed a recombination rate of 5.75 bins per Mb on average, which was significantly lower than that of the noninversion region of 6.84 bins per Mb (*t* test, *P* < 2e-16) (Fig. S9D). Moreover, the average recombination rate of 4.34 cM/Mb in the inversion region was significantly lower than that of 5.51 cM/Mb in the noninversion region (*t* test, *P* < *2*e-16) (Fig. S9D). These findings collectively provide compelling evidence supporting the association between inversions and the suppression of genomic recombination.

### The genetic architecture of heading date in the MAGIC population

The MAGIC population exhibited a varied heading date from 51.8 to 132.2 days and from 46.1 to 136.2 days in the two years (Fig. S10A). Among the founders, ZS97 displayed the earliest heading date, with 62.4 and 63.7 days, while XS134 exhibited the latest heading date, with 103.3 and 103.9 days. A strong positive correlation was observed between the heading dates recorded under the two environments (Table S10). The broad heritability was remarkably high, reaching 97.9%.

**Figure 4.**
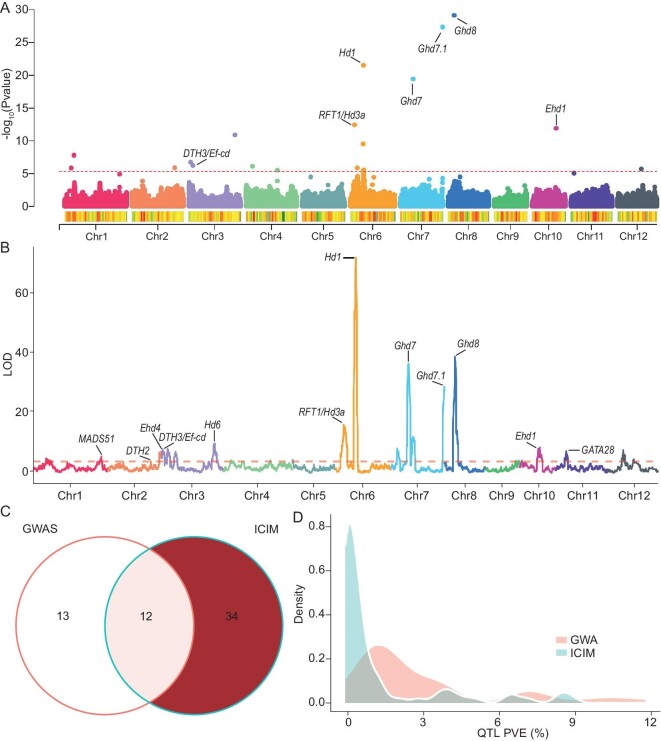
Genetic basis of heading date in the MAGIC population. A. Manhattan plots for heading date based on SNP GWAS. Known genes are marked, and the red dashed line represents threshold 4.4e^−6^. B. Distribution of LOD in linkage analysis of heading date. Known genes are marked, and the red dashed line represents threshold 4.0. C. Venn diagram of QTL between association analysis and linkage analysis. D. Comparison of phenotype variance explained by single QTLs identified by association analysis and linkage analysis.

The threshold for claiming QTLs for GWAS at the SNP level was determined as 4.4e-6 by the Benjamini and Hochberg approach. A total of 25 significant loci were identified to associate with heading date (Fig. 4A; Table S11). In addition, permutation tests (*n* = 1000, *P* < 0.05) showed that a threshold of LOD = 4.0 was set for claiming QTLs in the linkage analysis based on the ICIM model using 24 144 bins. A total of 47 QTLs were detected (Fig. 4B; Table S12). Twelve QTLs were commonly detected by both methods. Whereas 13 QTLs were exclusively detected through association analysis, and additional 34 QTLs were only identified by linkage analysis, including many minor QTLs (Fig. 4C, D). Among the QTLs detected by linkage analysis, 19 were consistently associated, while 28 were specific to a single year. The significant signals identified through GWAS accounted for 59.5% and 59.7% of phenotype variance, while the identified QTLs through linkage analysis explained 60.9% and 62.3% of phenotype variance, respectively (Fig. S10B).

In general, single QTLs explained varied parts of phenotype variance from 0.4% to 8.4%. The additive effects between the parental alleles with the largest and the smallest effects at a given QTL ranged from 1.9 to 9.9 d. These results indicated that both a few major and many minor QTLs together regulate heading date. Specifically, the major QTLs *Hd1* and *Ghd7* exhibited additive effects of 9.9 d and 9.6 d, and 8.4 d and 9.1 d, respectively and explained 8.4% and 8.5%, and 3.7% and 4.0% of phenotype variance.

### Mapping resolution dependent on genetic effects of QTLs

To assess mapping resolution in the MAGIC population, we introduced the concept of offset distance, which quantifies the genomic separation between a known gene and its associated lead bin in linkage analysis. No significant correlation was observed between the offset distance and the additive effects, phenotypic variance explained by QTL and logarithm of odds scores of QTLs (Fig. S11). The offset distance spanned a range from 0 to 211 kb, with an average of 94 kb, equivalent to 0–18 bins with an average of 6 bins (Table S13). The offset distances for major genes, such as *Hd1, Ghd7* and *RFT1*, were 39 kb, 55 kb and 0 kb in the MAGIC population, respectively, corresponding to 4, 3 and 0 bins. Minor genes such as *Ehd1, MADS51* and *DTH3* exhibited offset distances of 61 kb, 107 kb and 123 kb, respectively, corresponding to 4, 8 and 8 bins.

### New causal variations for heading date

A set of 21 heading date genes exhibiting natural variations has been isolated in rice [33]. Except for 2 genes, *Hd16* and *RCN1*, 19 genes showed functional polymorphisms among the eight parents (Table S14, Table S15). A total of 3 copy number variations and 35 nucleotide variations causing functional polymorphisms were identified in 19 genes, of which 15 nucleotide variations were newly discovered. Of them, 14 genes, including *MADS51, DTH2, Ehd4, Ef-cd, DTH3, Hd6, RFT1, Hd3a, Hd1, Ghd7, Ghd7.1, Ghd8, Ehd1* and *GATA28*, were in the regions of heading date QTLs detected in this study. In contrast, *OsHESO1, OsCOL4, Hd17, Hd18* and *MADS56* exhibited functional variation among the eight parents, but they were not identified in this study.

Two heading date genes, *GIC* and *SIP1*, isolated by the reverse genetic approach were in the confidence intervals of minor QTLs *qhd4.6* and *qhd9*, respectively (Figs S12A, S12B, S13A, S13B). *GIC*, affecting chloroplast development [34], may be the causal gene of *qhd4.6* because a frameshift variant was found in *GIC* alleles from three Xian rice varieties, ZS97, MH63 and 9311 (Table S16). In addition, this variation is associated with heading date across multiple environments in 529 germplasms (Fig. S12C). *SIP1* physically interacts with *OsTrx1* to promote the expression of *Ehd1* and facilitate flowering [35]. In three Xian rice varieties (ZS97, MH63 and 9311), two frameshift variants were identified in *SIP1* as candidate causal variants (Table S16). In the 529 germplasm resources, these two variations were associated with heading date across the four environments (Fig. S16). The expression levels of key downstream components related to heading date, including *Hd1, Ehd1, RFT1* and *Hd3a*, were upregulated in the accessions with two frameshifts (Fig. S15). A MAGIC line (MG863) with heterozygous 9311 and MITAK alleles at *SIP1* was identified. A near-isogenic F_2_ population of 60 plants from MG863 was constructed to evaluate the genetic effect of *SIP1* (Fig. S13C). The expression levels of downstream *Ehd1* and *Hd3a* were upregulated in the plants with the 9311 allele, while there was no significant difference in *RFT1* (Fig. S14). Indeed, the MITAK alleles significantly delayed heading by 5 d compared with the 9311 allele (Fig. S13D, S13E). Thus, the frameshift is the causal variation for heading date.

**Figure 5.**
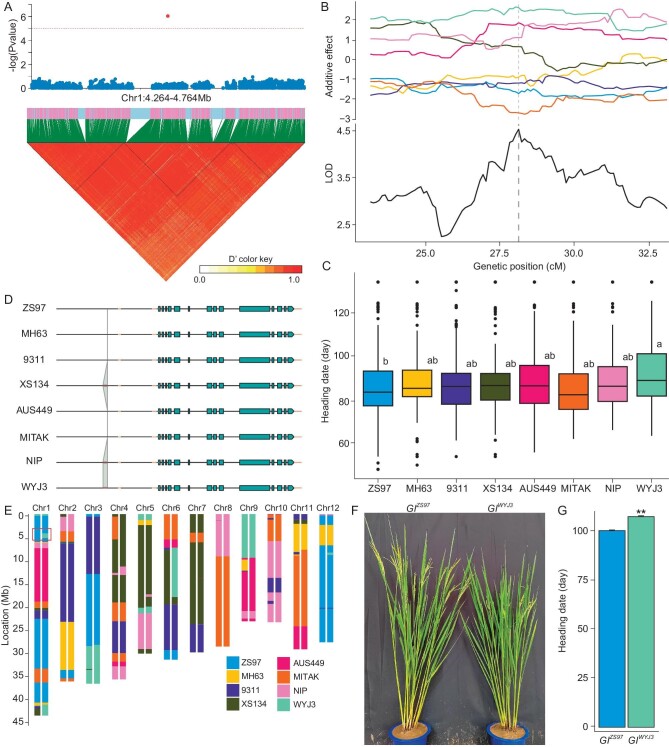
Structural variant in the *OsGI* promoter heading. A. Manhattan plot and LD block plot for heading date in the MAGIC population at the beginning of chromosome 1. B. Plot of the additive effect and LOD for heading date in the MAGIC population at the beginning of chromosome 1. The dashed line represents the QTL. C. Multiple comparisons of heading date among alleles of eight founders at lead bin locus. Different letters indicate significant difference at *P* < 0.05 level via Duncan test. The sample size per genotype is more than 58, see Table S17 for details. D. The structural variant in the *OsGI* promoter among the eight founders; yellow represents UTRs, and green represents exons. E. Genotype of the heterozygous inbred line for *OsGI*. F. Plant phenotype in near-isogenic lines of *OsGI*. G. Heading date in near-isogenic lines of *OsGI*, sample size for each genotype *n* = 10. ** represents significance at *P* < 0.01.

A significant signal cluster was consistently detected in the short arm distal on chromosome 1 in both years (Fig. 5A). The additive effects of the Geng rice alleles of XS134, NIP and WYJ3 were larger than those of the Xian rice alleles of ZS97, MH63 and 9311 (Fig. 5B). Moreover, a significant difference was detected between ZS97, WYJ3 and another six parental alleles (Fig. 5C). To identify the candidate gene, we compared the genome sequence of the target bin and identified a 220-bp deletion in the promotor of the circadian clock gene Os*GI* from parents ZS97, MH63, 9311, AUS449 and Mitak (Fig. 5D; Table S16). The 220-bp insertion/deletion located 477 bp upstream of the transcription start site was associated with early heading and increased expression level of *OsGI* in flag leaves before heading (Fig. S18). The 220-bp region has several ATTAAT and GTGGC motifs annotated as light responsive elements, and TATA-box annotated as important transcription regulator and a 51 nt uORF, while these motifs were previously reported to regulate *Ehd1* expression [36]. Additionally, the expression levels of key downstream components such as *Hd1, Ehd1* and *Hd3a* in the photoperiod flowering pathway showed noticeable enhancements in the germplasm resources with this deletion (Fig. S18). This genetic variation was associated with heading date in the germplasm resources (Fig. S19). Accordingly, its genetic effect was verified by the residual heterozygous line MG349 with heterozygous ZS97 and WYJ3 alleles at *OsGI* (Fig. 5E). The expression levels of *OsGI, Ehd1* and *Hd3a* were upregulated in the plants carrying the ZS97 allele, while there was no significant difference in *Hd1* and *RFT1* expression (Fig. S17). In fact, the WYJ3 allele significantly delayed heading by 7 d compared with the ZS97 allele (Fig. 5F, G).

### Genetic effect comparison among multiple parental alleles

To investigate the genetic effects of different parental alleles, we performed multiple comparisons for heading date genes identified in this study. For these 14 genes, *OsGI, Ehd4, Ef-cd, DTH3, Hd6, GIC, RFT1, Hd3a, Hd1, Ghd7, Ghd7.1, Ghd8, SIP1* and *Ehd1*, but not *MADS51, DTH2* and *GATA28*, more than three levels of genetic effects were demonstrated across all eight parental alleles (Table S17).

**Figure 6.**
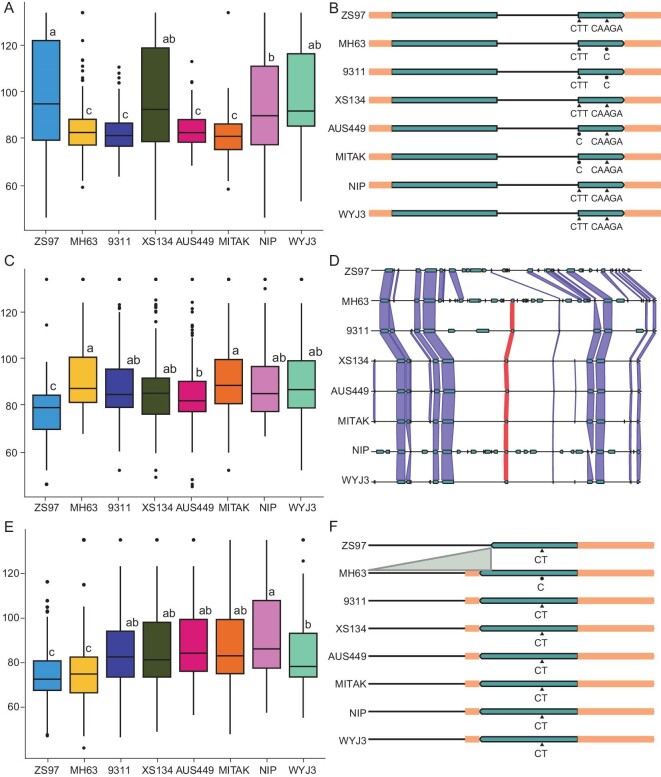
Comparison of genetic effects among parental alleles at three major heading date genes. Allele effects of *Hd1* (A), *Ghd7* (C) and *Ghd8* (E), and functional variants of *Hd1* (B), *Ghd7* (D) and *Ghd8* (F) in eight parents. Different letters indicate significant difference at *P* < 0.05 level via Duncan test. The y axis is heading date (day), and the sample size per genotype is more than 55, see Table S17 for details.

For *Hd1*, the founders ZS97, XS134, NIP and WYJ3 possessed complete gene coding sequences but with variations, while MH63 and 9311 displayed a 4-bp deletion in exon 2, and AUS449 and MITAK exhibited a 2-bp deletion in exon 2, resulting in loss of function (Fig. 6B). The lines possessing nonfunctional alleles of MH63, 9311, AUS449 and MITAK had heading dates of 83 d. Compared with the nonfunctional alleles, the NIP allele had a weak effect of delaying heading by 9 d, the ZS97 allele had a strong effect of delaying heading by 17 d, and the alleles of XS134 and WYJ3 had a moderate effect of delaying heading by 14 d between the ZS97 and NIP alleles (Fig. 6A). These results revealed that the functional alleles of *Hd1* delayed heading dates. Regarding *Ghd7*, ZS97 completely lacked the gene, while the remaining seven parents possessed the complete gene coding sequence (Fig. 6D). The lines possessing the ZS97 allele had a heading date of 78 d, the AUS449 allele had a weak effect of delaying heading by 8 d, the 9311, XS134, NIP and WYJ3 alleles had a moderate effect of delaying heading by 12 d, and the MH63 and MITAK alleles had a strong effect of delaying heading by 15 d (Fig. 6C). Thus, *Ghd7* alleles had 4 levels of genetic effects among 8 founders. In the cases of *Ghd8* and *Ghd7.1*, four and five levels of genetic effects were identified among the eight parents (Fig. 6E, F; Fig. S20A, S20B). More than three magnitudes of alleles were identified for the remaining 10 genes (Table S17).

## DISCUSSION

### The eight-way MAGIC population is an all-in-one population for QTL mapping

Xian and Geng rice exhibit significant phenotypic and genetic differences due to their distinct ecological niches and environmental adaptations. Therefore, when the intersubspecies mapping population is used for QTL mapping, more genes are expected to be identified. The eight founders of the MAGIC population originate from diverse subgroups or ecotype regions, resulting in significant genetic and phenotypic variations. Approximately 44 000 genes showed functional polymorphisms among the eight founders, including variations in the coding sequence that led to missense mutations, frame shifts and premature stop codons. If considering the variations in regulatory regions such as *cis* elements, enhancers and silencers, more genes showed functional polymorphisms among the parents. This highlights the substantial genetic diversity captured by the population. Germplasm resources are expected to have more functionally diversified genes and map more QTLs than MAGIC populations. However, few genes have been identified; in particular, most of the known genes have not been identified in germplasm resources [37,38]. This is mainly caused by population structure, kinship, and the low frequency of novel functional variations. However, these functional variations can be effectively identified in the MAGIC population by GWAS because no distinct population structure exists. In addition, even if one rare allele in nature is only coincidently possessed by one parent, its frequency in the MAGIC population is ∼1/8 and is maintained for data analysis. Like the type-B response regulator gene *Ehd1*, the natural functional variation only existed in the AUS449 allele, but it was identified in the MAGIC population. Here, 10 known heading date genes were identified by GWAS, which is much more than the 4 known heading date genes identified in a germplasm of 950 accessions and 3 known heading date genes identified in a germplasm of 529 accessions [8,10].

The biallelic SNP-based GWAS was widely used to map QTLs with MAGIC populations, which often results in false negatives [20]. In contrast, the multiallelic bin approach circumvents this limitation because of more refined classification of the MAGIC lines, thus allowing for high sensitivity in detection. Once a bin map was constructed, linkage analysis was performed with the MAGIC population. Linkage analysis resulted in 47 QTLs, which is much more than the 28 QTLs identified with GWAS in this study. Linkage analysis identified 14 known heading date genes and 2 novel genes, of which 7 genes, *GIC, SIP1, DTH2, Hd6, Ehd4, MADS51* and *GATA28*, were not identified through GWAS. Only 5 known genes, *Hd1, Ghd7, Ghd7.1, Ghd8* and *Ehd1*, were repeatedly identified through GWAS in both years, while 14 known genes, except *GATA28*, were repeatedly detected by linkage analysis. More powerful QTL detection with multiple allele haplotypes was also found in both Drosophila and maize multiparental populations [20,39]. It is noted that only 5 of 19 heading date genes with functional polymorphisms among eight parents failed to be identified using linkage analysis in the MAGIC population. Most likely, these undetected genes are sensitive to a very long day length because they were originally identified in high latitudinal regions and experimental controlled environments, where the day length is 14.5 hours in the summer solstice, approximately half of one hour longer than that of Wuhan [40–44].

It is noticed that the segregation distortion is widely identified in the MAGIC population due to the reproductive isolation between Xian and Geng subspecies. Segregation distortion frequently happens in the regions linked to hybrid fertility genes and this influences the resolution and power of QTL mapping [45,46]. Recently, four genes *S5, f5, Pf12* and *Sc* together ensure the fertility of intersubspecies hybrids [47,48]. To avoid the distortion in a population derived from an intersubspecies hybrid, these four genes are suggested to be edited or replaced in some selected parents before used for crossing so that these loci are fixed with Xian or Geng alleles or without the killer genes in all parents.

### Gradually changing genetic effects of multiple alleles provide flexibility for genetic improvement

In the past, genetic effects of important genes were estimated in biparental populations. Like our previously isolated heading date gene *Ghd7*, we compared its genetic effects on heading date between two parental alleles. When we estimated the effects for multiple alleles, we developed a series of near isogenic lines in the same background and compared the phenotype. Then, three types of alleles were screened with three levels of genetic effects, including nonfunctional alleles, weak alleles, and strong alleles [49]. Here, rich allelic diversity existed in the MAGIC population, which provides a good chance to estimate the genetic effects of all eight parental alleles at the bin level. Here, four types of *Ghd7* alleles had gradually changing genetic effects. In addition to our previously reported three types, a new type of allele carried by AUS449 possessed intermediate genetic effects of an 8-day delay (Table S11). In a previous study, *Hd1* was predominantly categorized into two discrete genetic effects: functional and nonfunctional. Here, the functional alleles could be further classified into weak functional alleles from NIP, which delay heading by 9 days; moderate alleles from XS134 and WYJ3, which delay heading by 14 days; and strong functional alleles from ZS97, which delay heading by 17 days (Fig. 6A). For the other genes *Ehd4, Ef-cd, DTH3, Hd6, RFT1, Hd3a, Hd1, Ghd7, Ghd7.1, Ghd8* and *Ehd1*, the parental alleles have varied genetic effects spanning multiple levels (Table S11). In most cases, japonica rice carries weak alleles, and indica rice carries either strong alleles or nonfunctional alleles.

Rice plant is sensitive to photoperiod. Short-day length promotes heading. There are several rice production zones in China, in which there are different rice cropping seasons such as one cropping season in northeast and north of China and two cropping seasons in the Yangtze River basin. The day length is getting shorter and shorter from north to south during rice growing season and the varieties in these regions gradually become more sensitive to day length [31,49,50]. Thus, for these major heading date genes, alleles with strong, intermediate, weak, and nonfunctional effects should be reasonably deployed to the varieties in these cropping regions because they have distinct photoperiod sensitivity. Namely, the strong alleles are privileged to be selected for varieties in the south of China which is rich in light and temperature resources. Weak alleles are pyramided in the varieties in the north of China with moderate resources, and nonfunctional alleles act in the varieties in northeast of China with poor resources. On the other hand, the plants with the different allele-combinations probably have equivalent photoperiod sensitivity and resultant similar heading dates. Thus, these alleles identified in this study provide flexibility in enhancing the adaptability of rice varieties to diverse ecological regions.

### A high recombination rate contributes to precise QTL mapping in the MAGIC population

In the progress of developing the MAGIC population, three generations of outcrossing and subsequent selfing generations reorganized the genetic composition of eight parents. The eight-way MAGIC population is expected to exhibit a higher number of recombination events compared to other populations. This population comprises 24 144 bins with an average bin length of 15 kb, and 130 976 recombination events were identified in the population, indicating that a qualitative gene would be mapped to a bin of 15 kb and that a QTL would be mapped to several bins covering hundreds of kb regions in theory. For example, genes with large effects, such as *Hd1, Ghd7* and *RFT1*, were mapped to a 50-kb region, and genes with minor effects, such as *Ehd1, MADS51* and *DTH3*, were mapped to a 120-kb region.

In summary, at least 80% of the genes were functional polymorphisms among the eight parents, which provides a good chance to identify many genes in the MAGIC population. The results of both GWAS and linkage analysis confirmed that the MAGIC population has powerful QTL detection because of no distinct population structure and multiple allele comparison. Major QTLs could be mapped with a high resolution of 100 kb. Therefore, the MAGIC population is encouraged to map the QTLs for agronomic, physiologic, and developmental traits measured by advanced high-throughput phenotyping platforms.

## MATERIALS AND METHODS

Detailed materials and methods are available in the supplementary information.

## Supplementary Material

nwae222_Supplemental_File

## Data Availability

All raw reads generated for the individuals in the MAGIC population and the genomes of four parents have been deposited in the National Genomics Data Center under BioProject PRJCA021567.
