# Supplementary material for: Powerful QTL mapping and favorable allele mining in an all-in-one population: a case study of heading date
Source: Natl Sci Rev. 2024 Jun 26;11(8):nwae222. doi: 10.1093/nsr/nwae222 (PMC11360186; doi:10.1093/nsr/nwae222)
Supplement: nwae222_Supplemental_File [file nwae222_supplemental_file.zip › supFigure.docx]

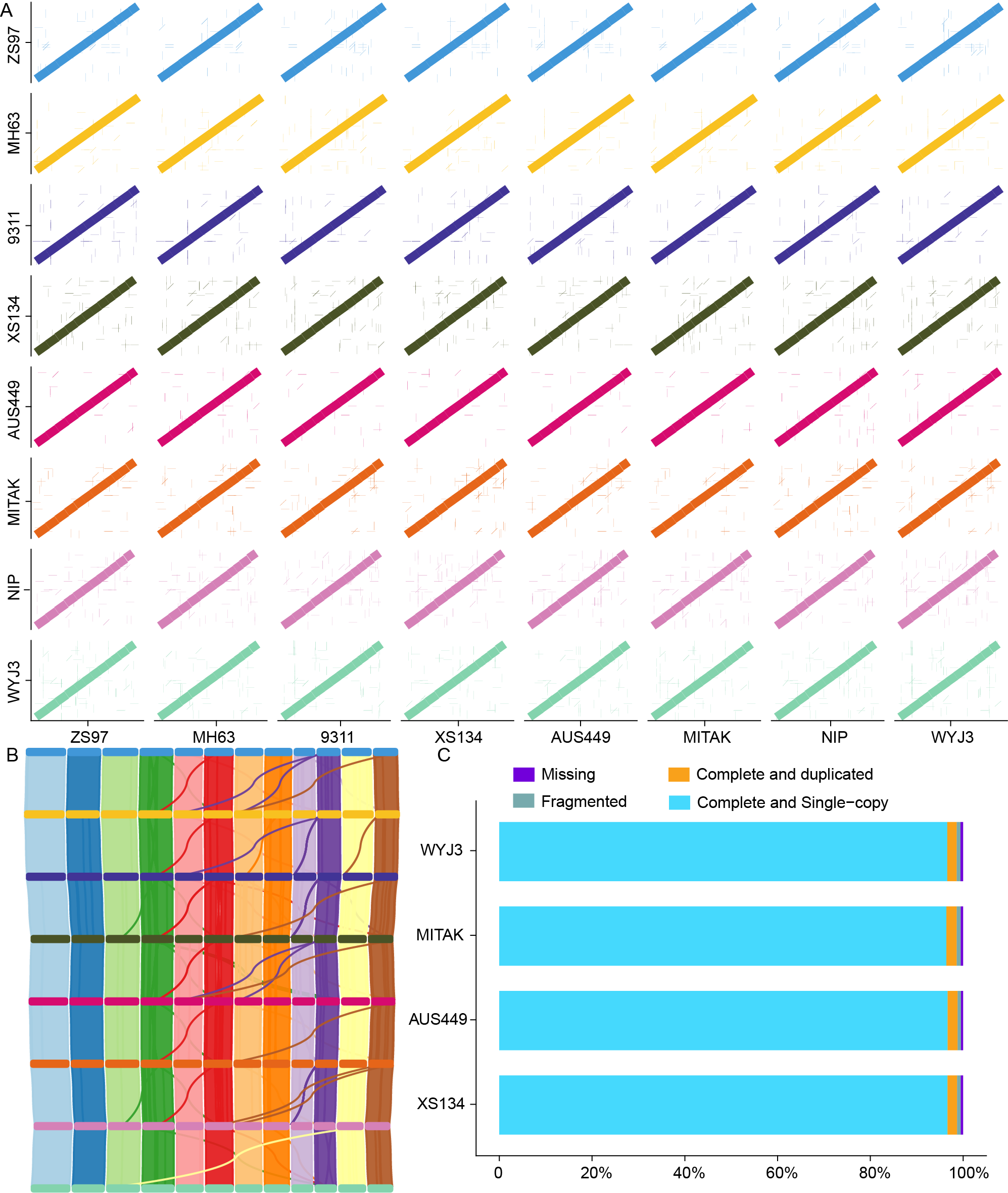


**Supplementary Fig. 1** The genome of eight parents

**A**. The paired dotplot plot among eight parents. **B**. Genomic synteny among eight parents. **C**. BUSCO evaluation of four novel genomes.


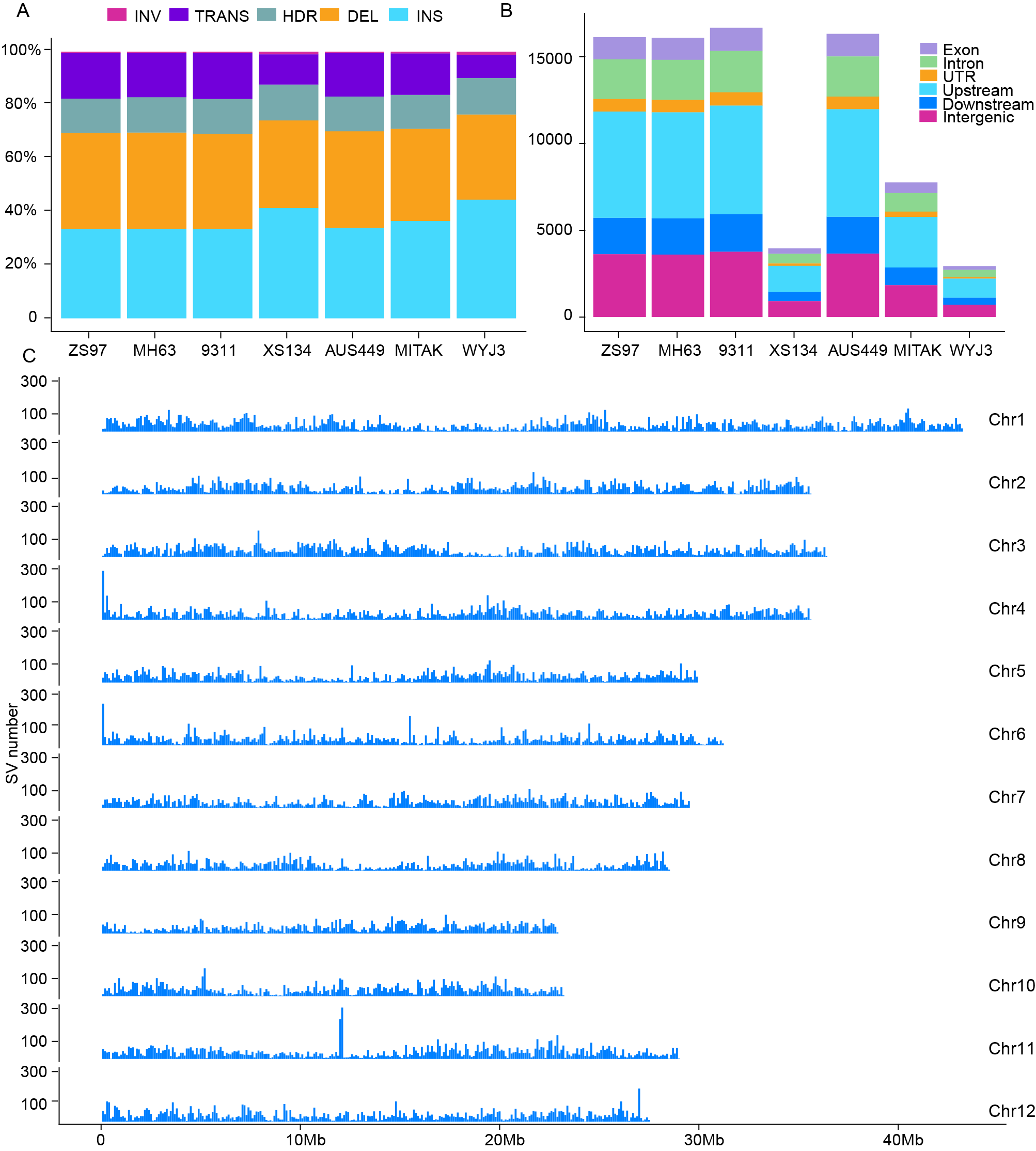


**Supplementary Fig. 2** The structure variants among 7 parents

**A**. The ratios of various structure variants in the 7 parents, INV represents inversion, TRANS represents translocation, HDR represents high diversity region, DEL represents deletion, INS represents insertion. **B**. Number of different annotation of structure variants for 7 parents. **C**. Distribution of structure variants on chromosome.


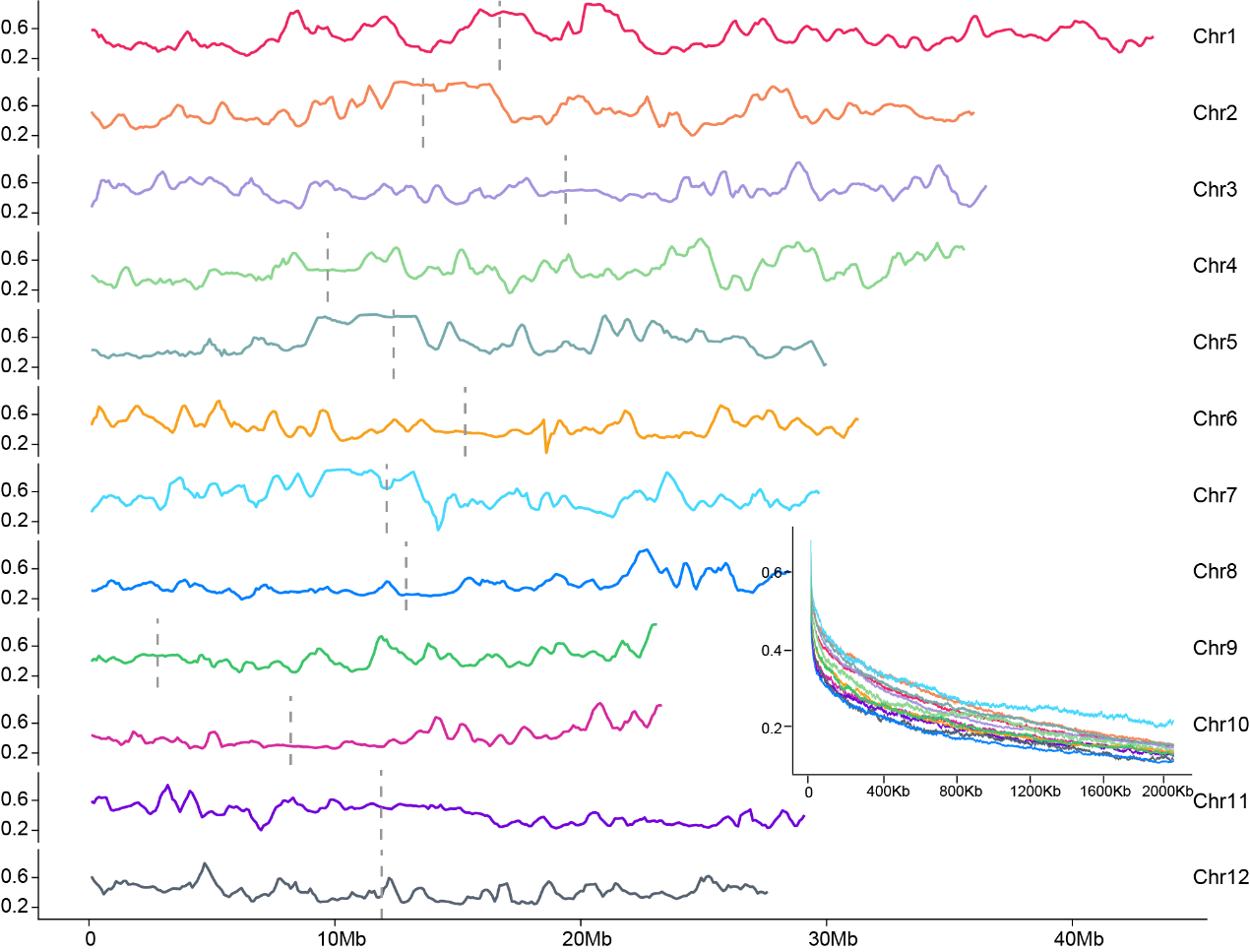


**Supplementary Fig. 3** Genome-wide LD pattern and LD decay plots of the MAGIC population


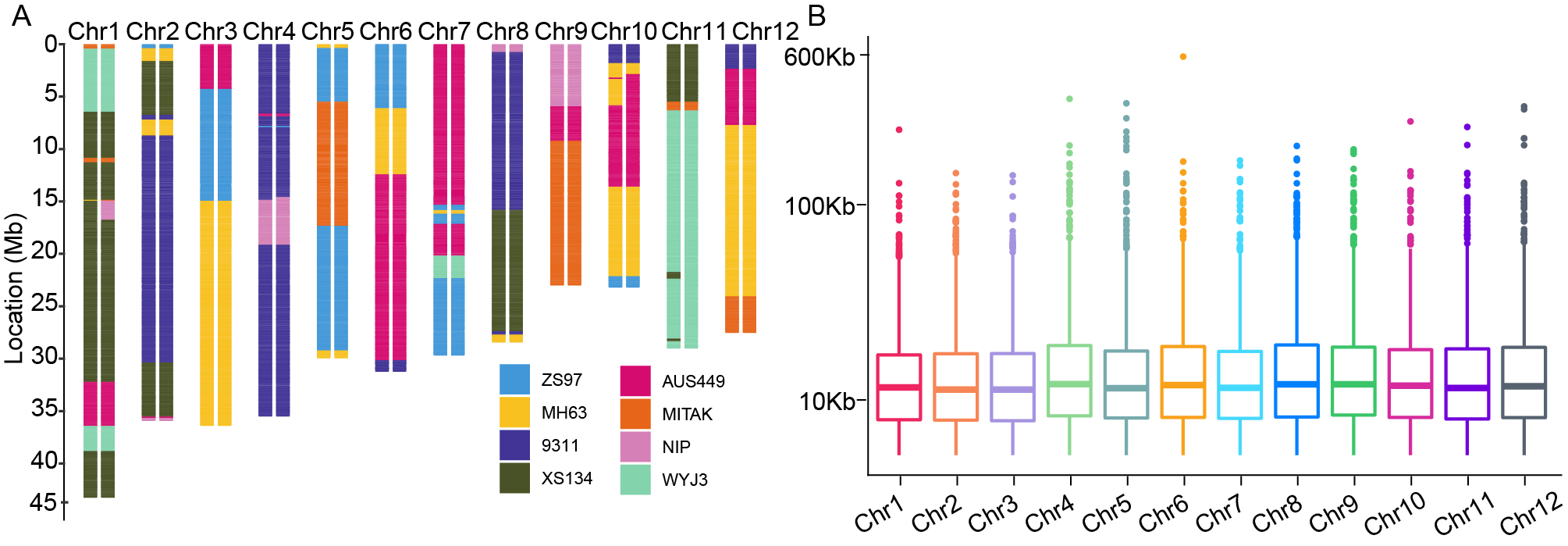


**Supplementary Fig. 4 Haplotype map of line MG4 and bin length across 12 chromosomes at the population level.**

**A**. Haplotype map of line MG4. **B**. Boxplot of bin lengths across 12 chromosomes at the population level.


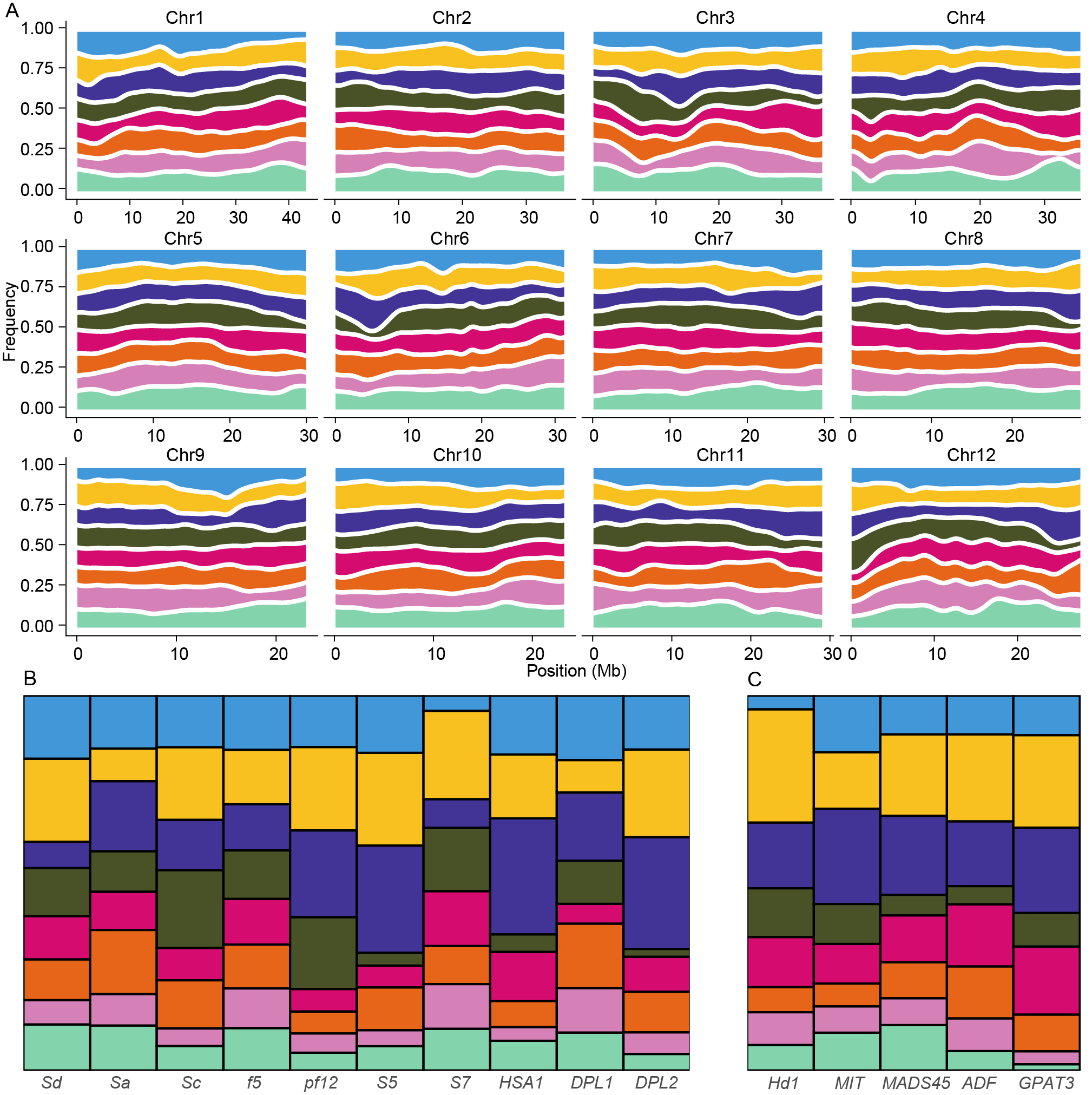


**Supplementary Fig. 5 Segregation distortion analysis of the MAGIC population**

**A**. Contribution of the eight founders across 12 chromosomes. The x-axis represents the physical position on chromosomes, and the y-axis represents the different ratios among the eight parents. **B**. The ratio of eight founders in the genetic incompatibility loci between Xian and Geng subspecies. **C**. The ratio of eight founders in the loci associated with male and female gamete development as well as flowering.


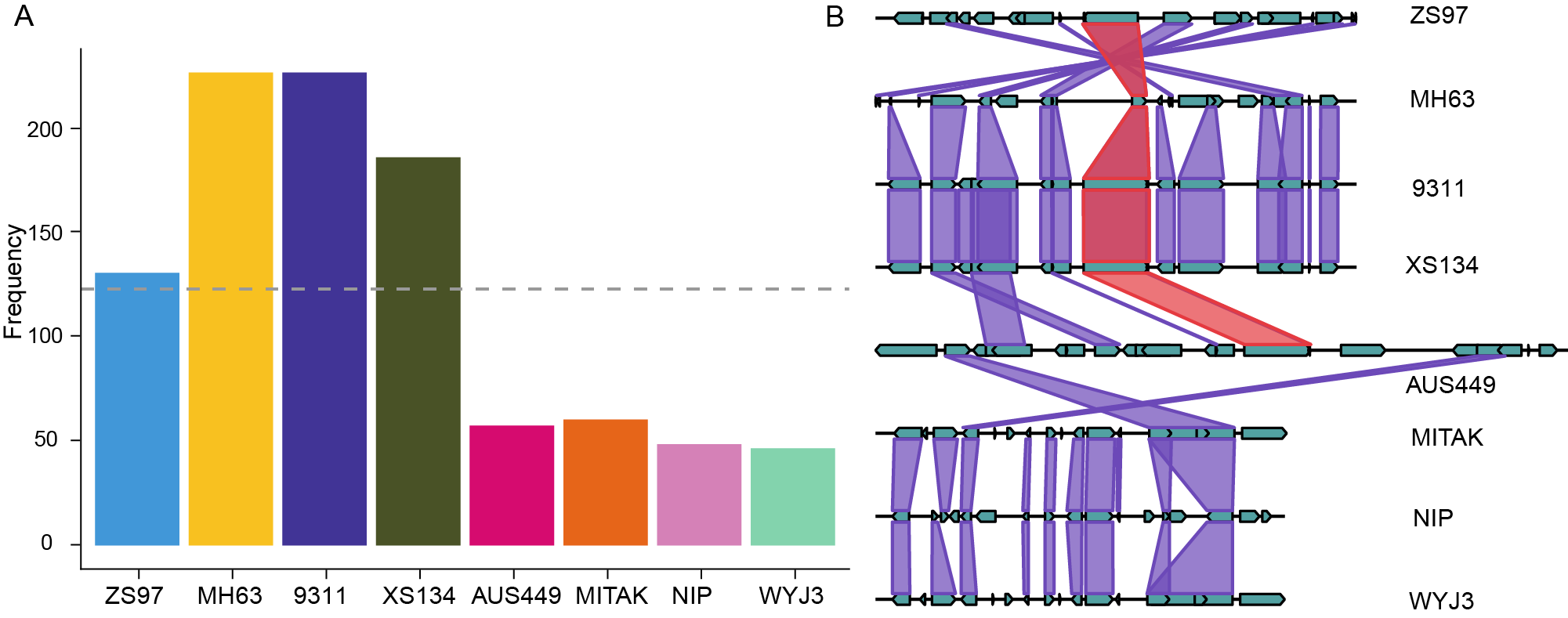


**Supplementary Fig. 6** Segregation distortion of male gamete hybrid sterility gene *pf12*

**A**. Frequency distribution of the *pf12* alleles of eight parents in the MAGIC population. **B**. Genetic collinearity of *pf12a* among eight parents.


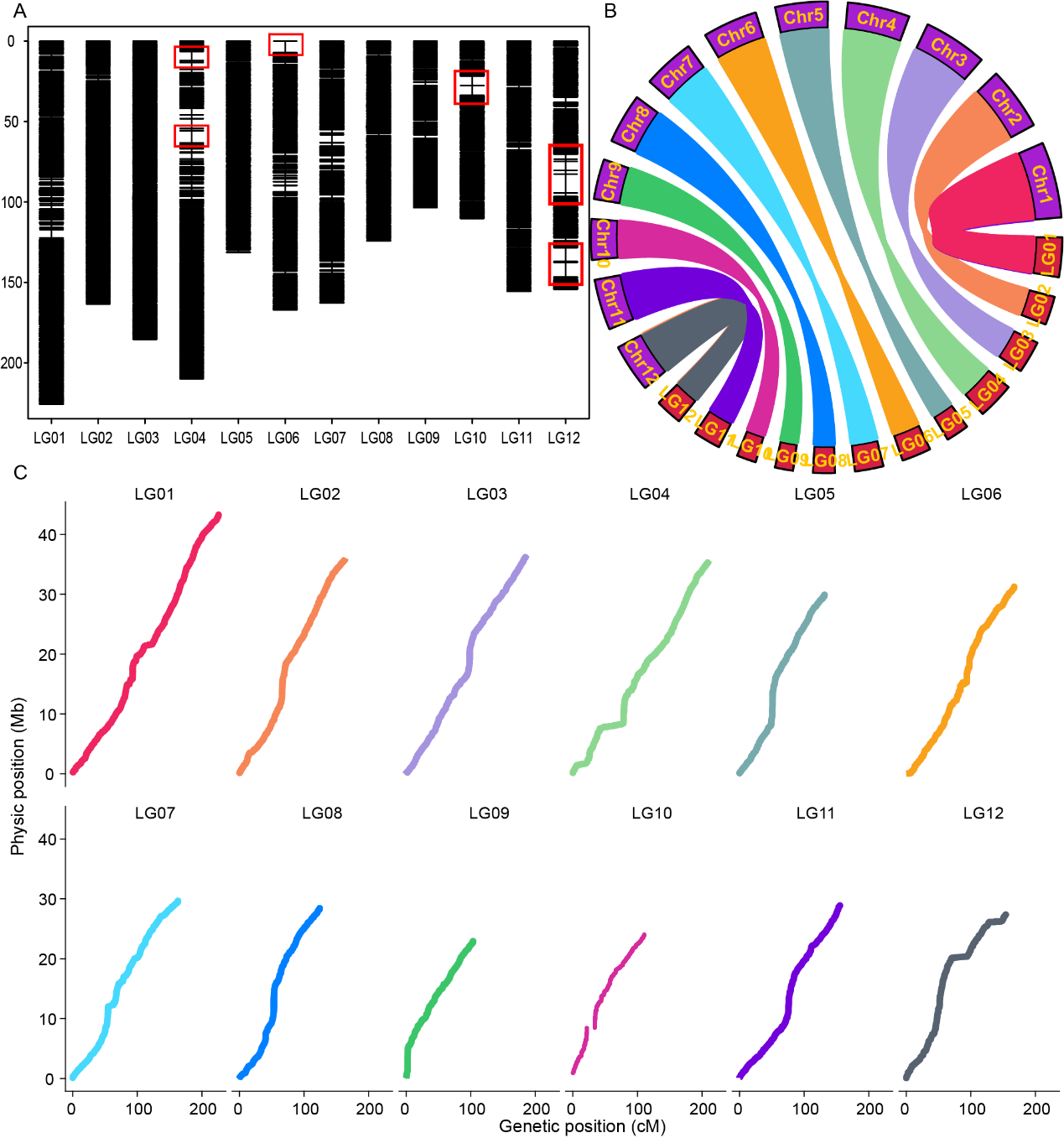


**Supplementary Fig. 7** Genetic linkage map construction of the MAGIC population

**A**. Genetic linkage map of the MAGIC population. **BC**. Collinearity of linkage map and physical map of MAGIC population.


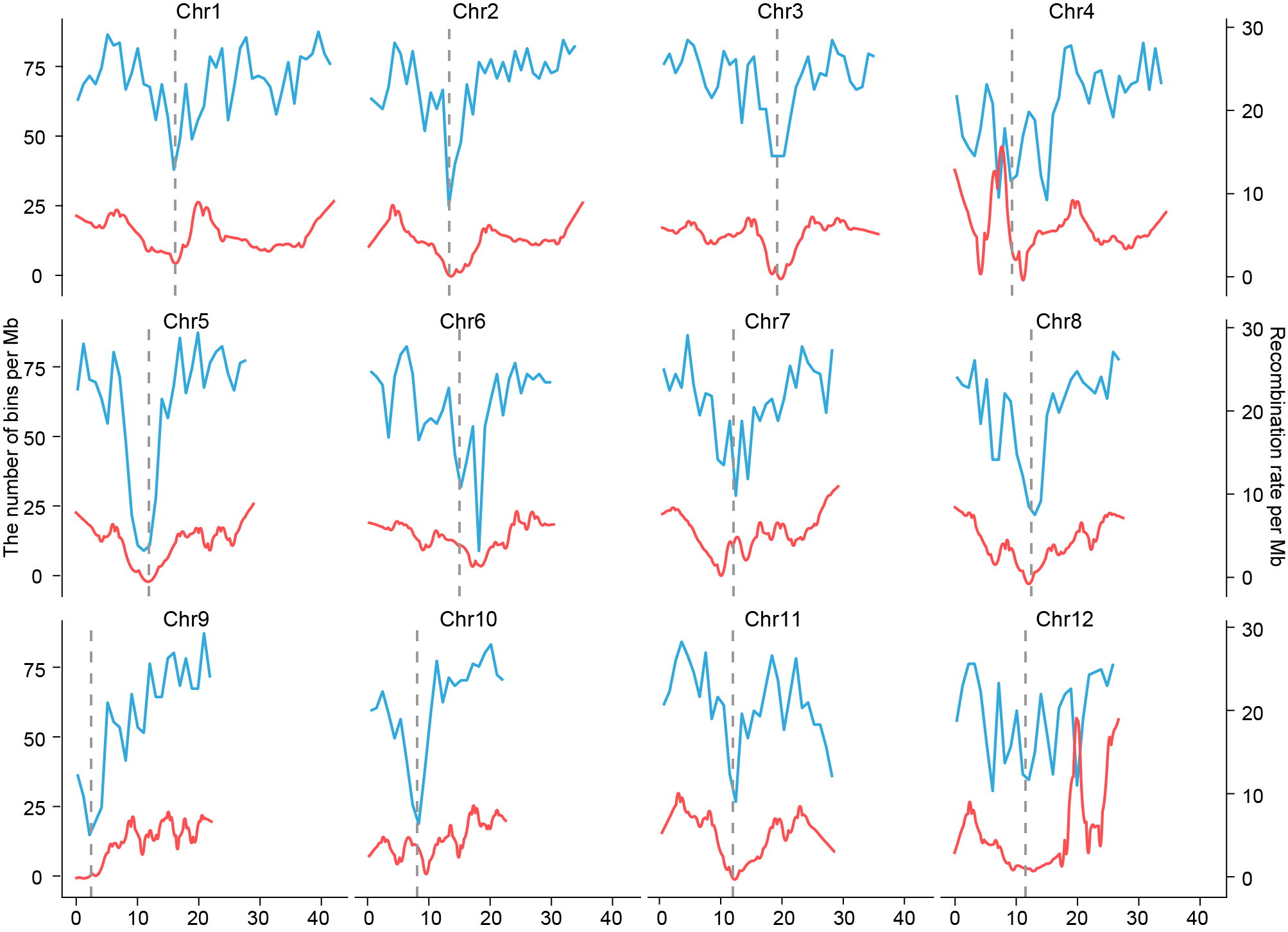


**Supplementary Fig. 8** The number of bins and recombination rate per Mb across 12 chromosomes

Dashed lines represent the positions of centromeres. Blue represents the distribution of the number of bins per Mb in the whole genome, and red represents the distribution of the recombination rate per Mb in the whole genome.


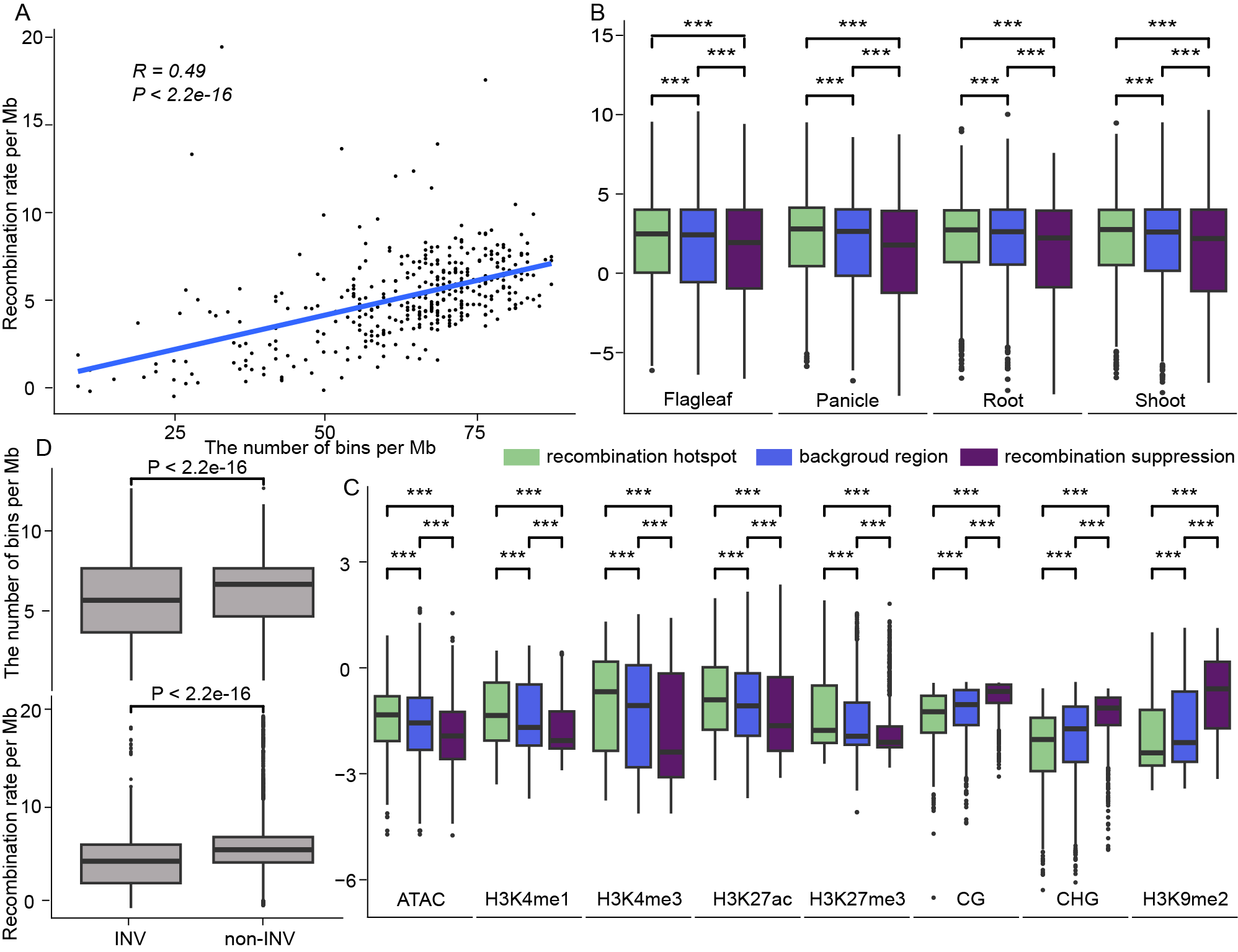


**Supplementary Fig. 9** Recombination across the whole genome in the MAGIC population

**A**. Correlation between the number of bins and recombination rate per Mb. **B**. Comparison of gene expression in four different tissues between recombination hotspots, recombination suppression and background regions. The y-axis represents the natural logarithm of TPM. **C**. Comparison of chromatin accessibility, histone modification and DNA methylation between recombination hotspots, recombination repression and background regions. The y-axis represents the natural logarithm of depth. **D**. Comparison of the number of bins per Mb and recombination rate per Mb between inversion and noninversion regions.


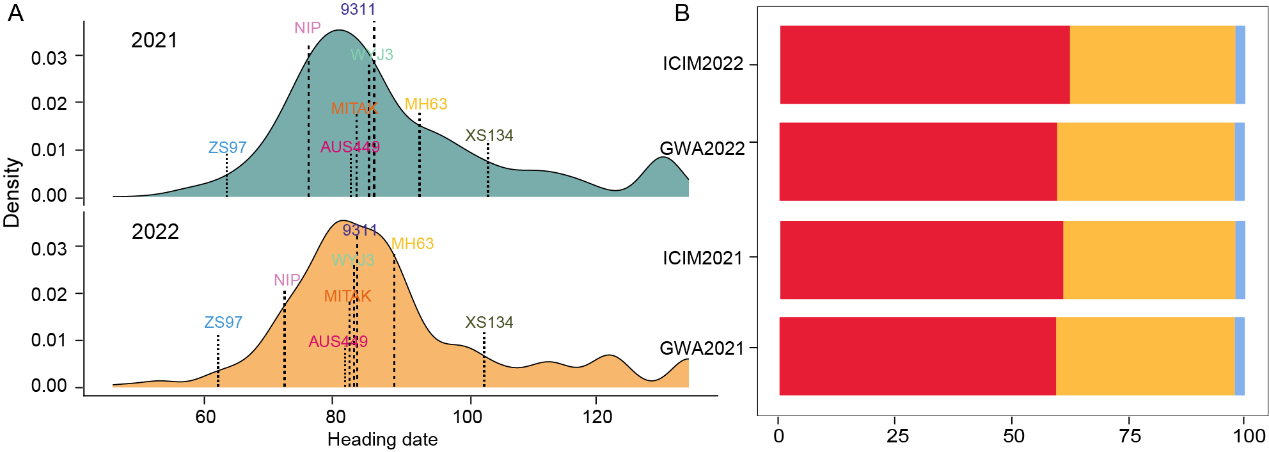


**Supplementary Fig. 10** Genetic mapping of heading date in MAGIC population

**A.** Heading date distribution in two years. **B.** Broad-sense heritability of two-year heading date data and missing heritability calculated by association analysis and linkage analysis, respectively. Red is the sum of genetic contribution of all QTLs, yellow is the missing heritability, which is the partial of heritability unexplained by QTLs. Red plus yellow is broad-sense heritability, and blue represents the ratio of environmental variance.


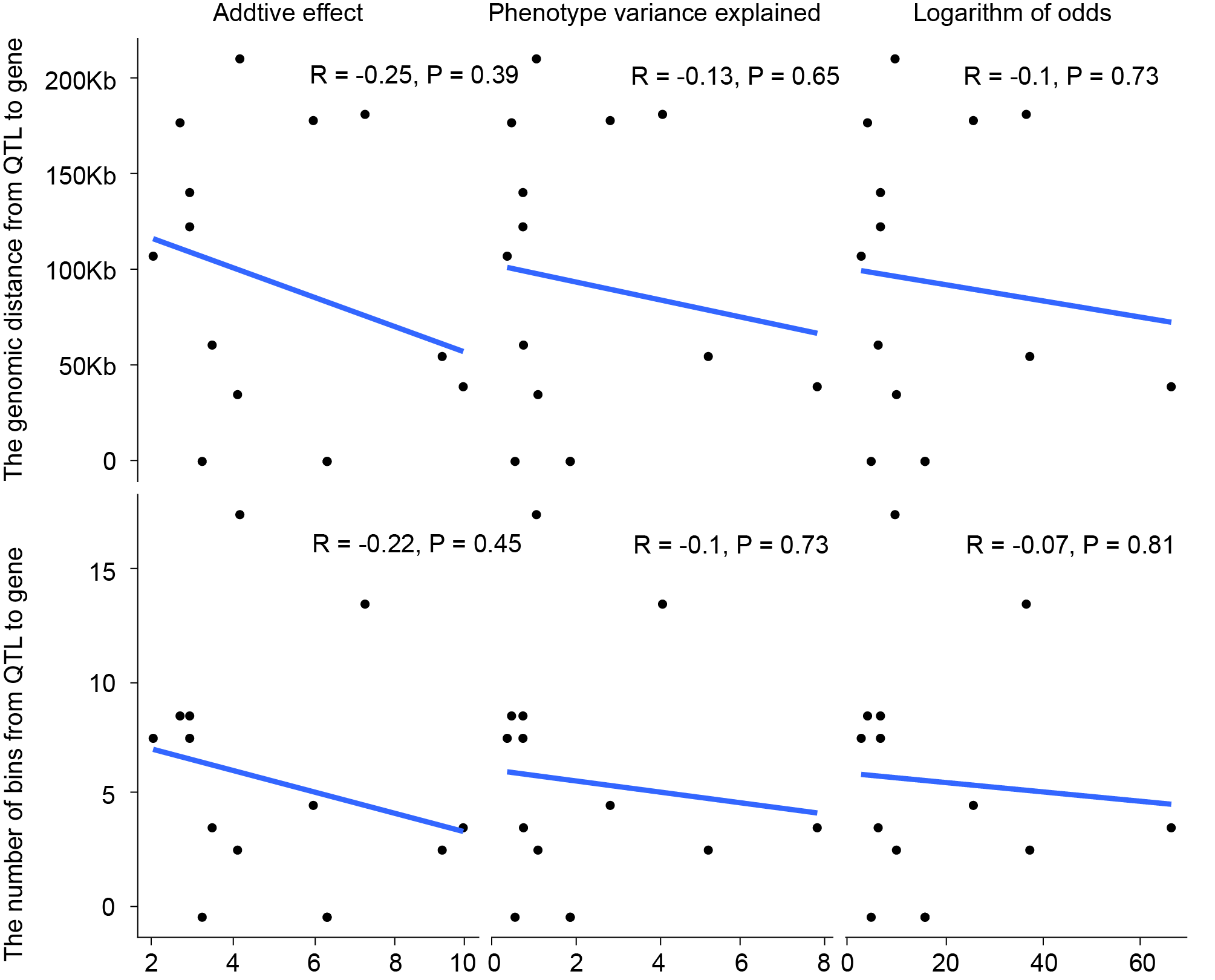


**Supplementary Fig. 11** Correlation analysis of offset distance of known genes

The y-axis is offset distance and offset bin number respectively, and the axis is additive effect, phenotype variance explained and LOD respectively.


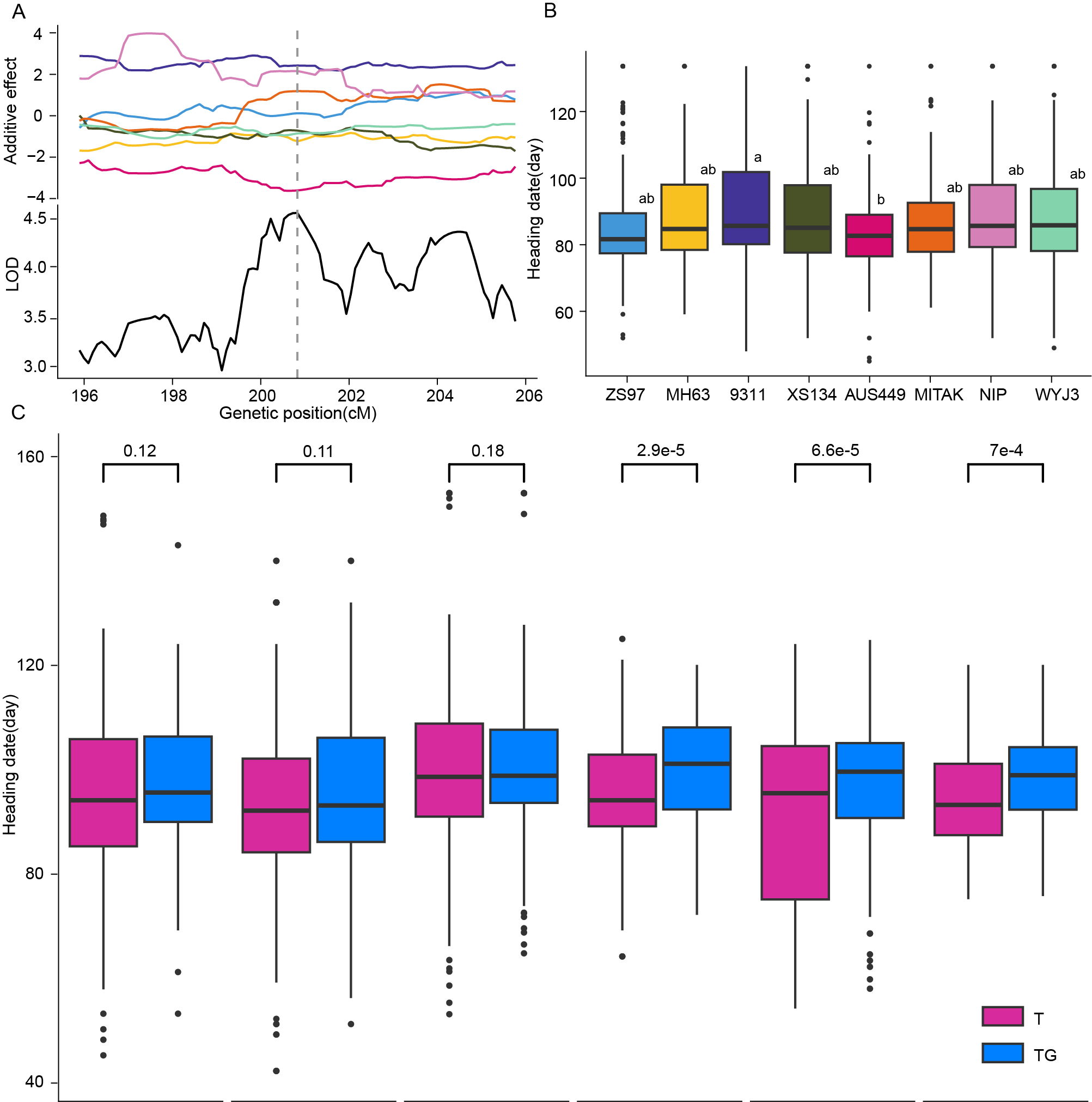


**Supplementary Fig. 12** *GIC* affects heading date.

**A**. QTL scanning in the interval of *GIC*. **B**. Multiple comparisons of heading date in eight alleles of *GIC*, the sample size per genotype is greater than 49, see Table S17 for details. **C**. Association of causal variation in *GIC* with heading date in six environments in germplasm resources. The first three environments are natural long day conditions in Wuhan, the last three are natural short day conditions in Lingshui. The sample size per genotype is greater than 100.


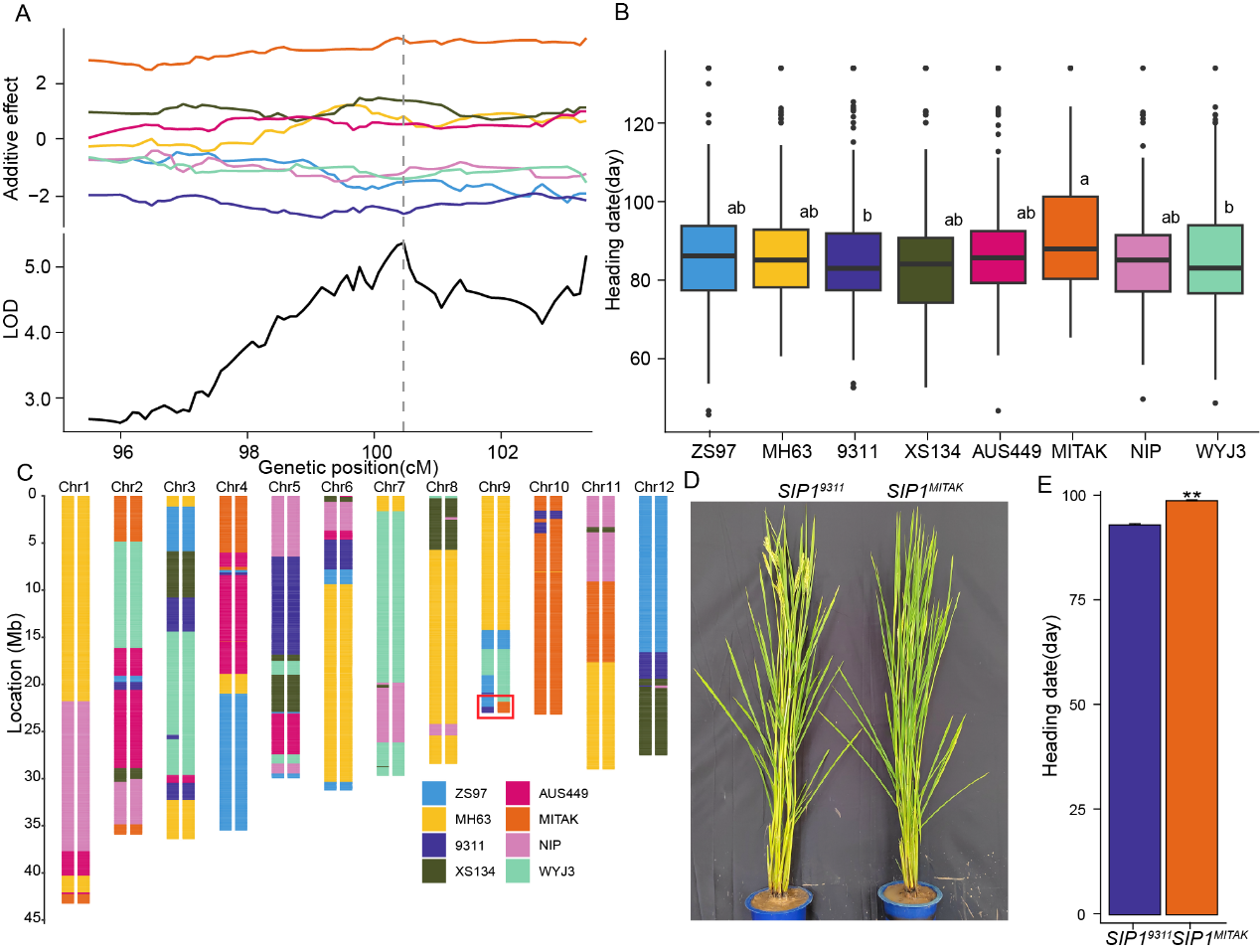


**Supplementary Fig. 13** *SIP1* affects heading date

**A**. QTL scanning in the interval of *SIP1*. **B**. Multiple comparisons of heading date in eight alleles of *SIP1*. **C**. Genotype of the heterozygous inbred line for *SIP1*, the sample size per genotype is greater than 83, see Table S17 for details. **D**. Plant phenotype in near-isogenic lines of SIP1. **E**. heading date in near-isogenic lines of *SIP1*, sample size n=10. ** represents *P < 0.01*.


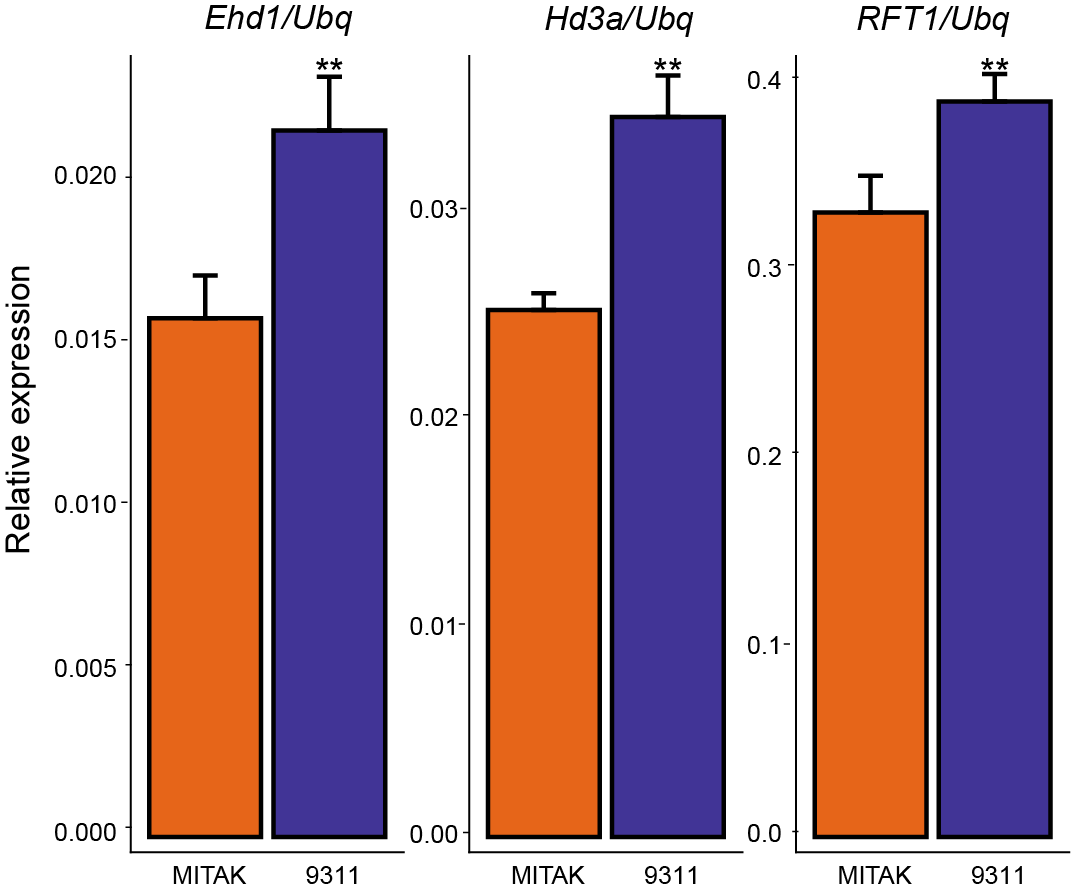


**Supplementary Fig. 14** Expression levels of *Ehd1*, *Hd3a* and *RFT1* in near-isogenic lines

Sample size n=3. ** represents *P < 0.01*.


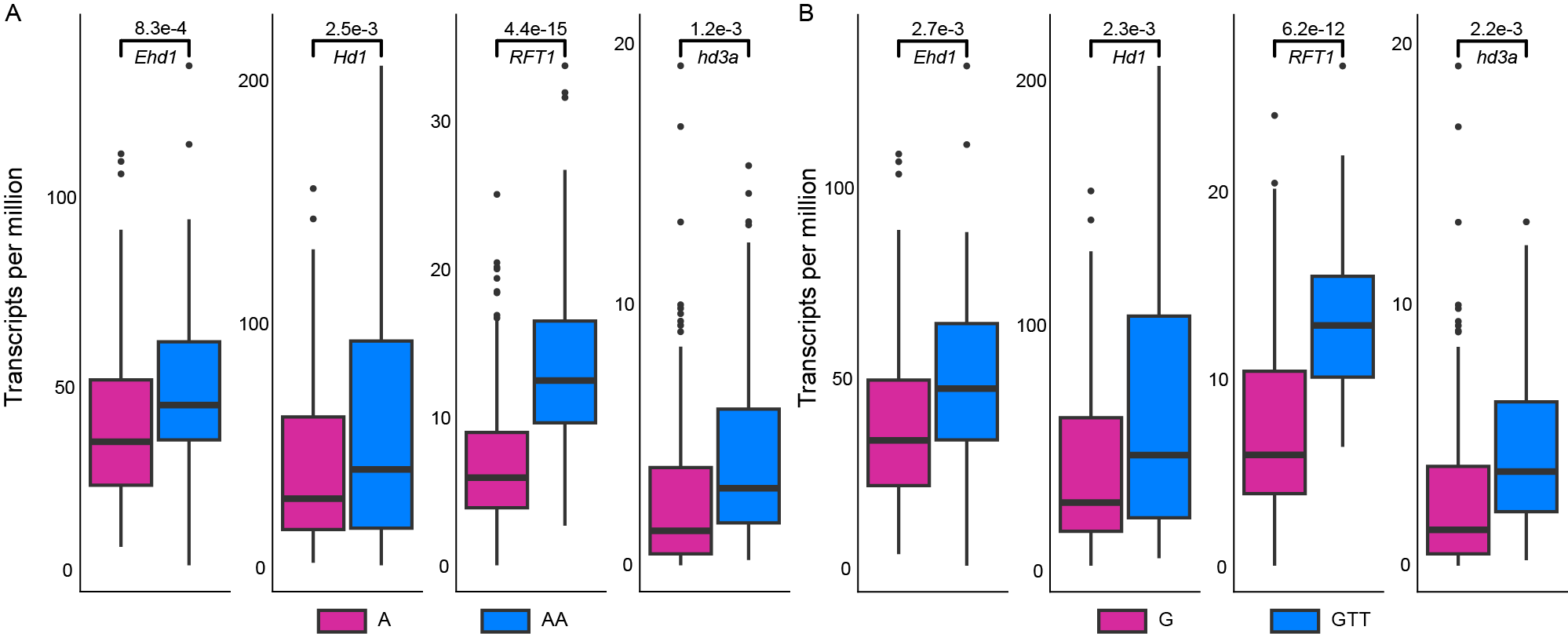


**Supplementary Fig. 15** Association of the expression levels of heading date gene *Hd1*, *Ehd1*, *RFT1* and *hd3a* with the two causal variations in *SIP1* in germplasm resources

(A) and (B) are different variants, the sample size per genotype is greater than 30.


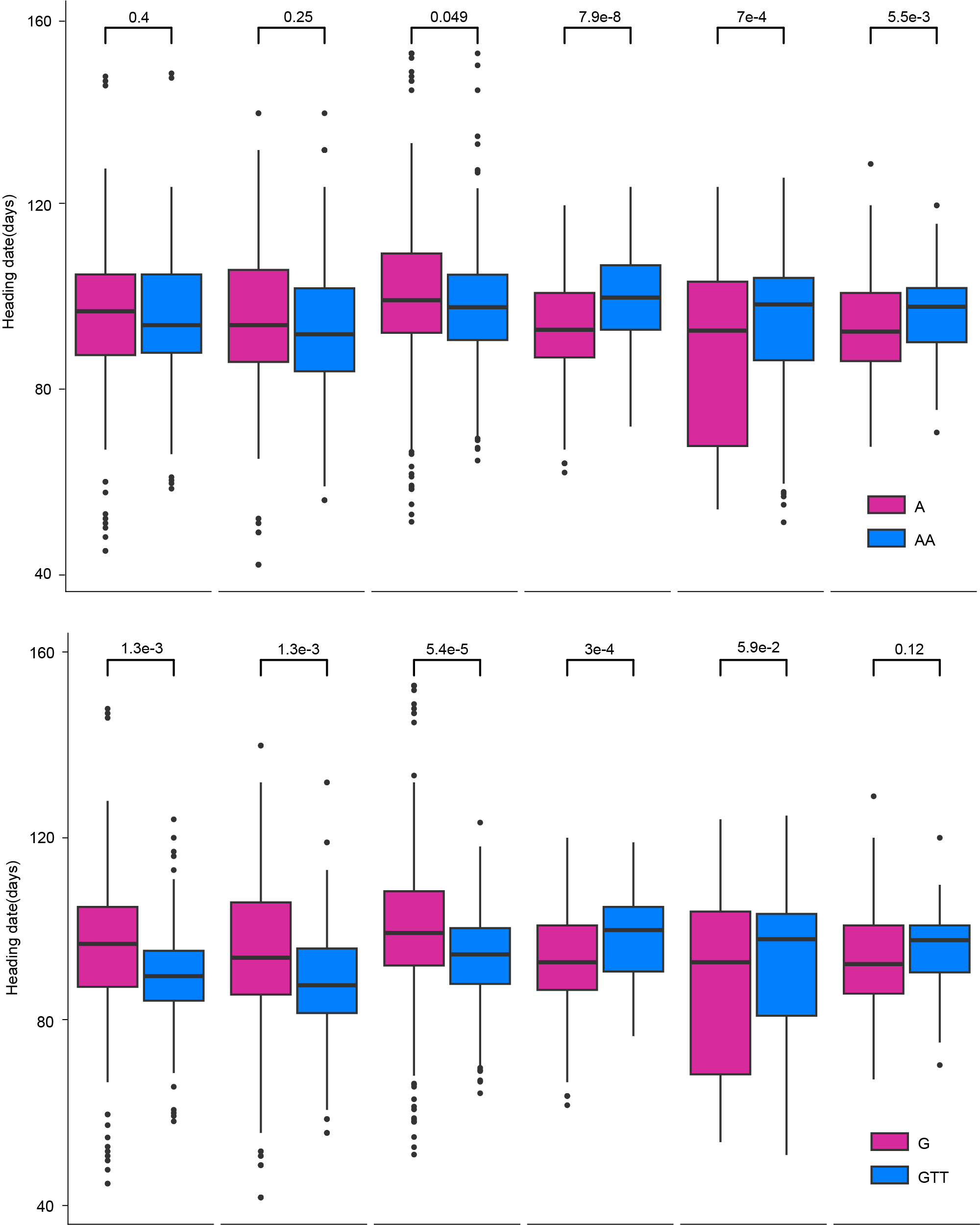


**Supplementary Fig. 16** Associations of the two causal variations in *SIP1* with heading date in six environments in germplasm resources

The sample size per genotype is greater than 100.


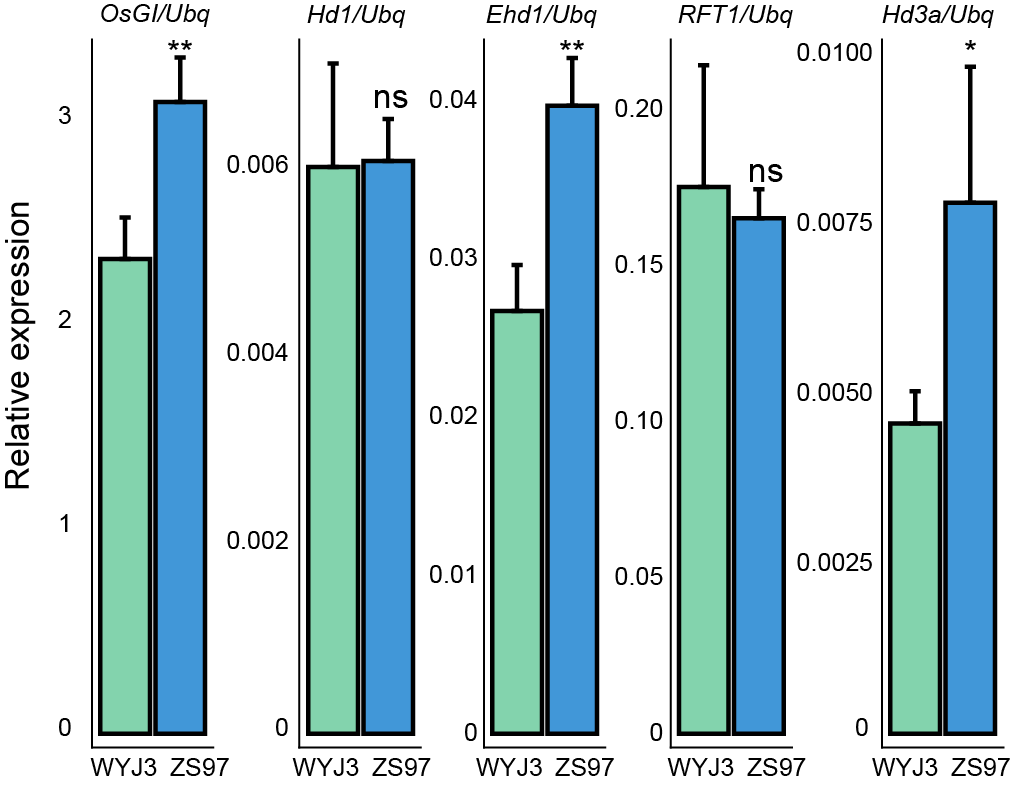


**Supplementary Fig. 17** Expression levels of *OsGI*, *Hd1*, *Ehd1*, *Hd3a* and *RFT1* in near-isogenic lines

Sample size n=3. Ns represents no significance. * represents *P < 0.05*, ** represents *P < 0.01*.


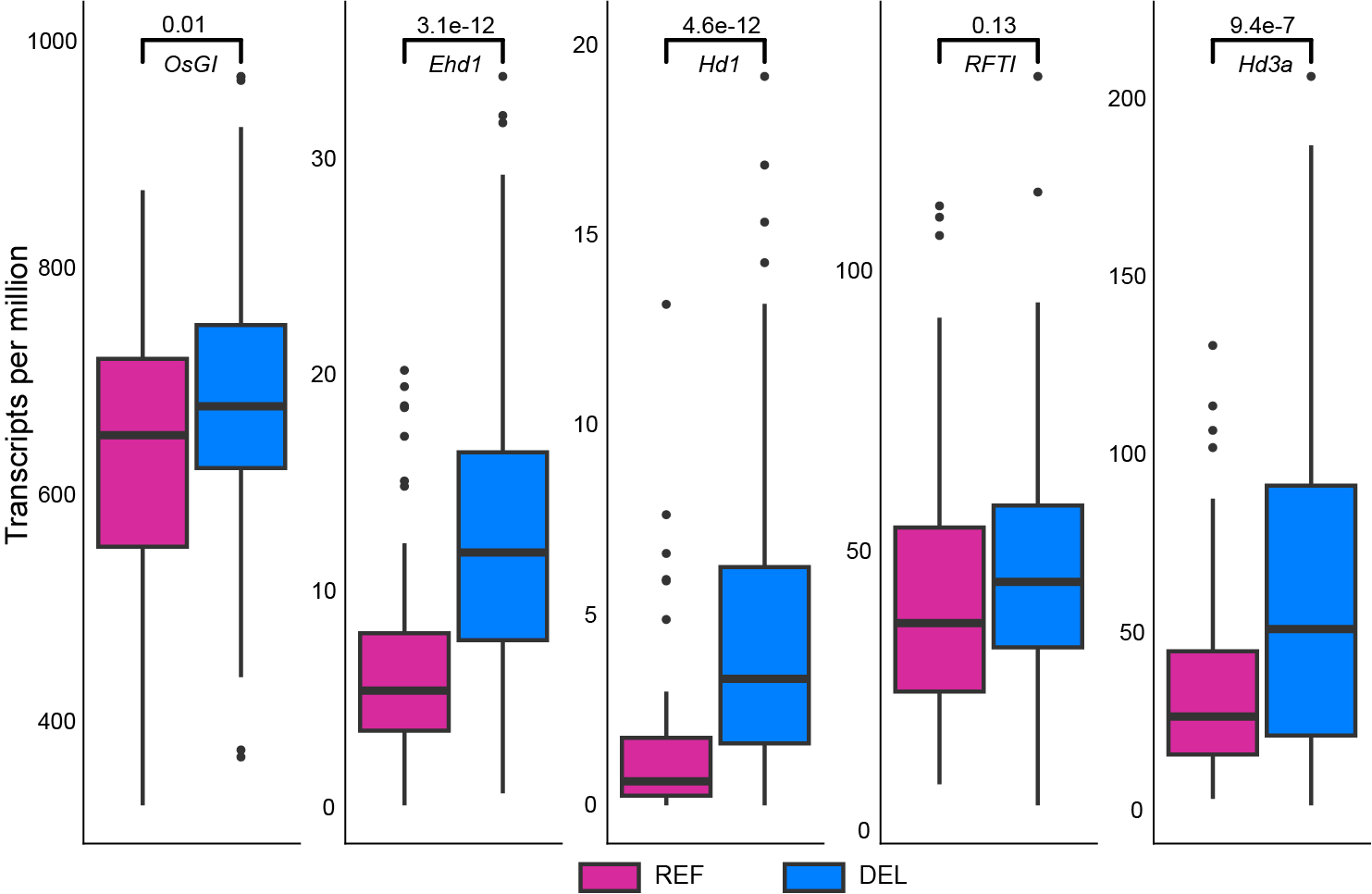


**Supplementary Fig. 18** Association of the expression levels of heading date gene *OsGI*, *Ehd1*, *Hd1*, *RFT1* and *Hd3a* with the structural variant of *OsGI* in germplasm resources

The sample size per genotype is greater than 30.


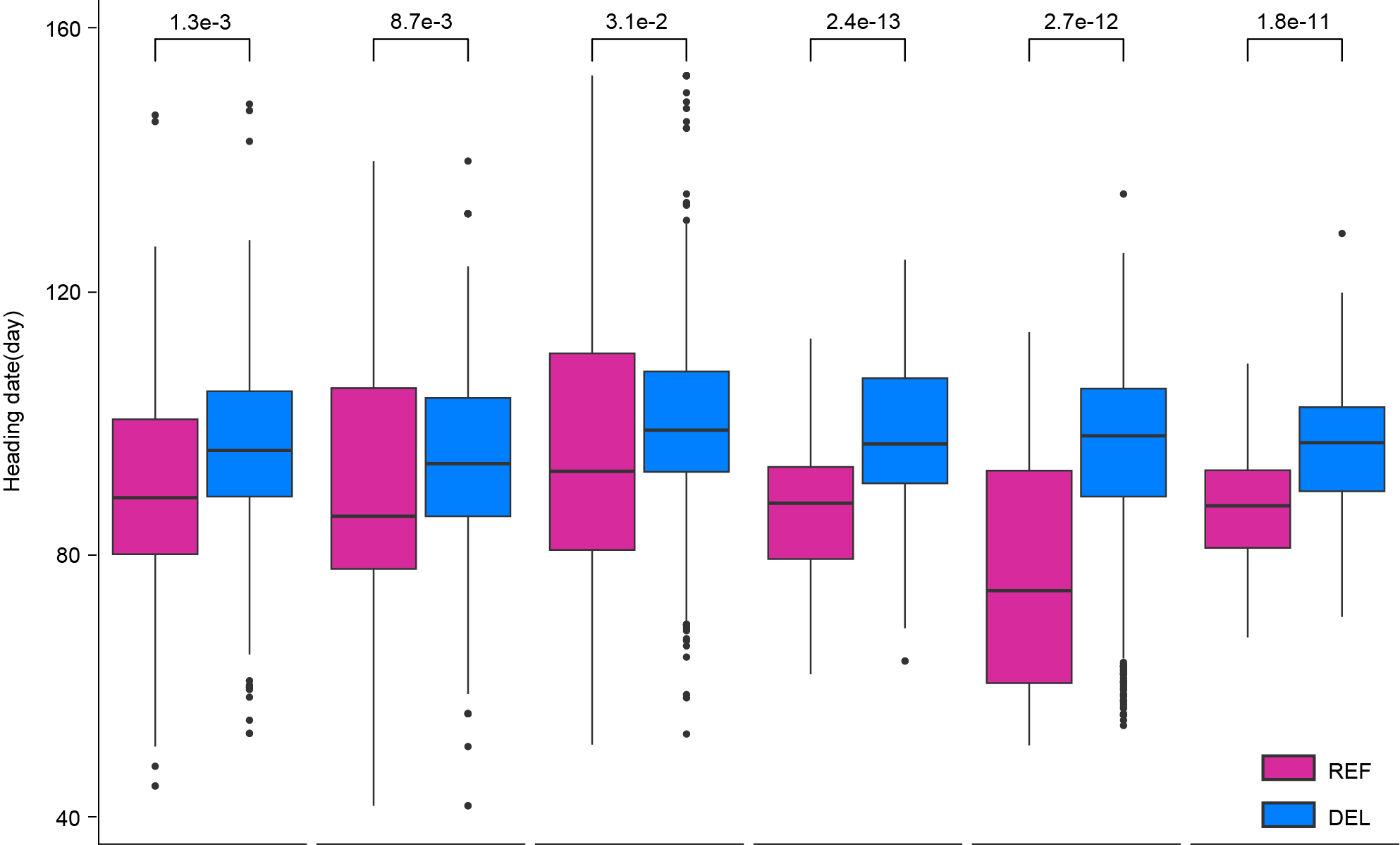


**Supplementary Fig. 19** Associations of the structural variant of *OsGI* with heading date in six environments in germplasm resources

The sample size per genotype is greater than 100.


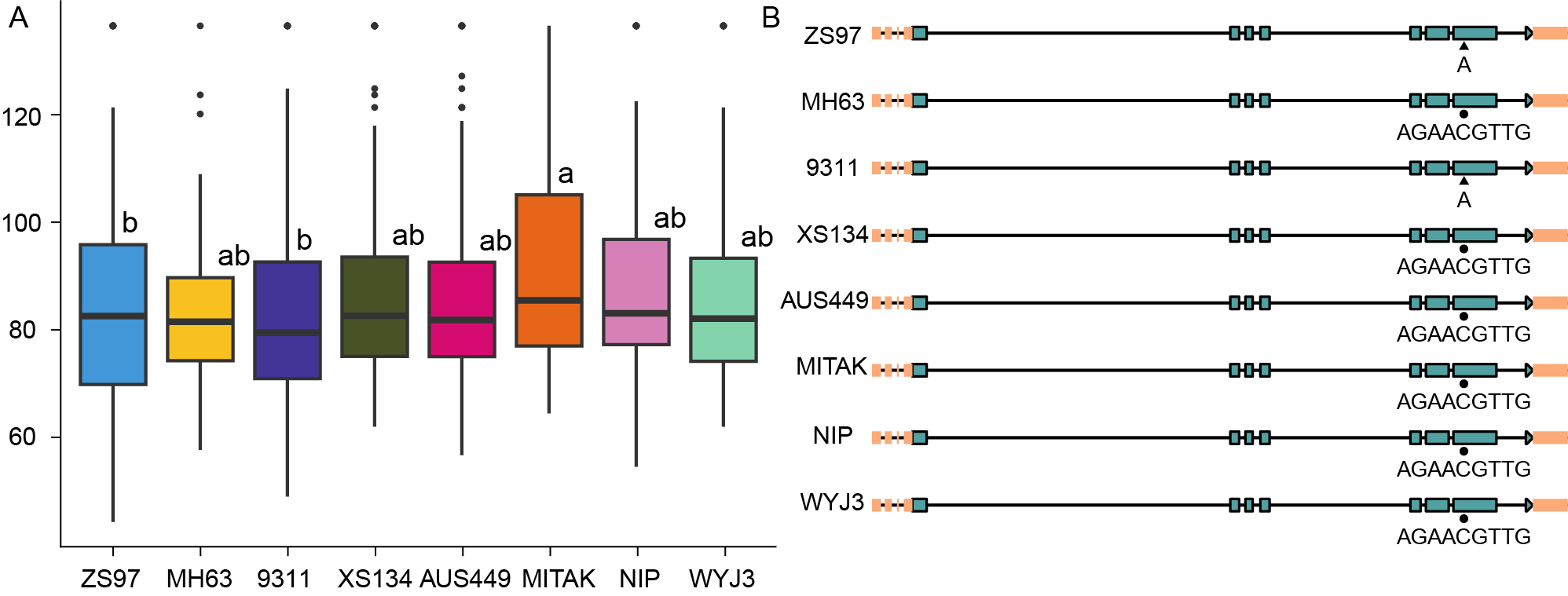


**Supplementary Fig. 20** Comparison of genetic effects among parental alleles at *Ghd7.1*

**A**. Allele effect of *Ghd7.1* in the MAGIC population. Different letters indicate significant difference at *P < 0.05* level via Duncan test. The y axis is heading date (day), and the sample size per genotype is more than 55, see TableS17 for details. **B**. Functional variants of *Ghd7.1* in eight parents.


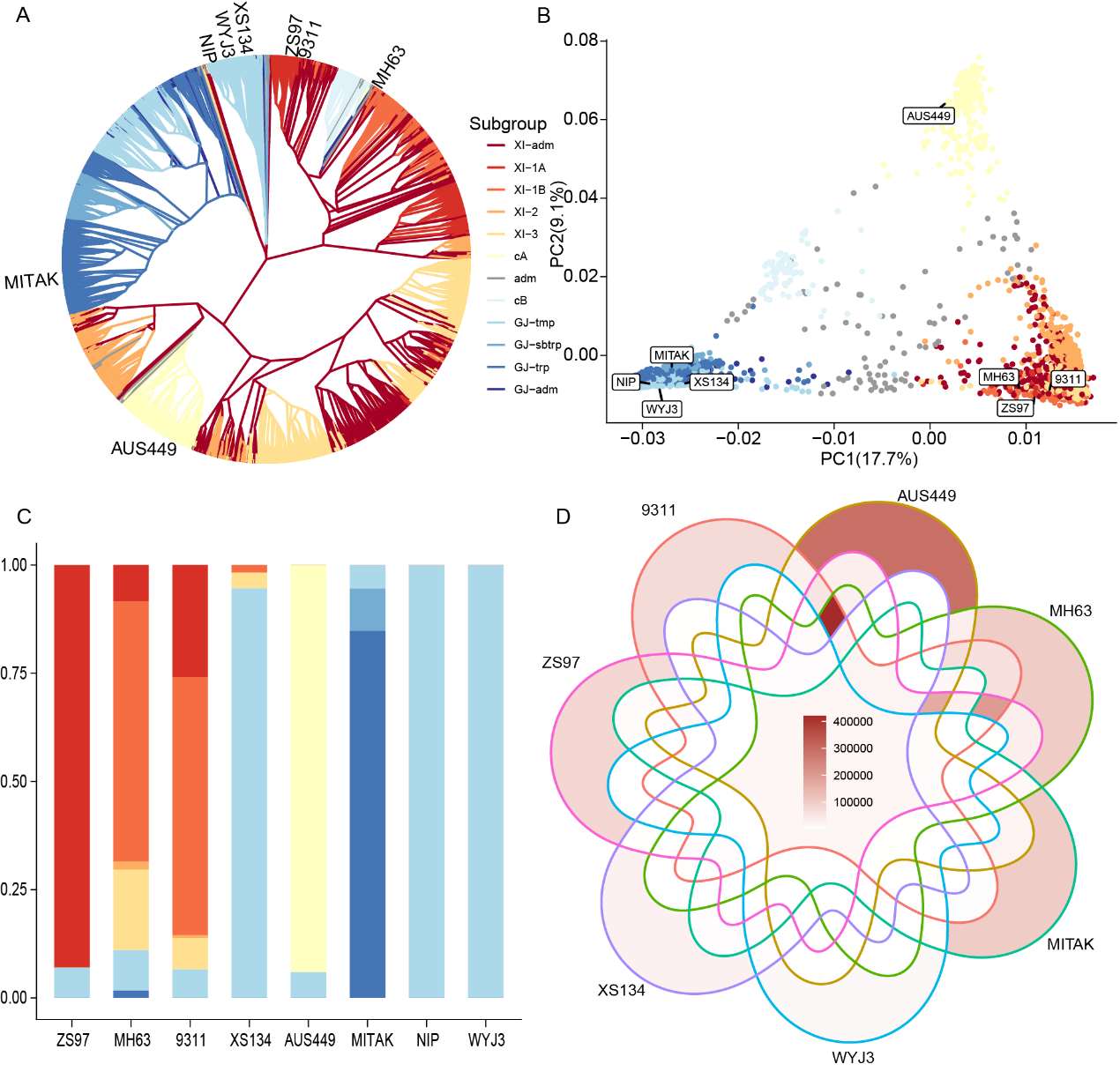


**Supplementary Fig. 21** population structure of eight founders

**A**. Phylogenetic analysis of eight parents in Rice3K. **B**. Principal component analysis of eight parents in Rice3K. **C**. Ancestry composition analysis of eight parents in Rice3K. **D**. SNP variation diversity of eight parents.


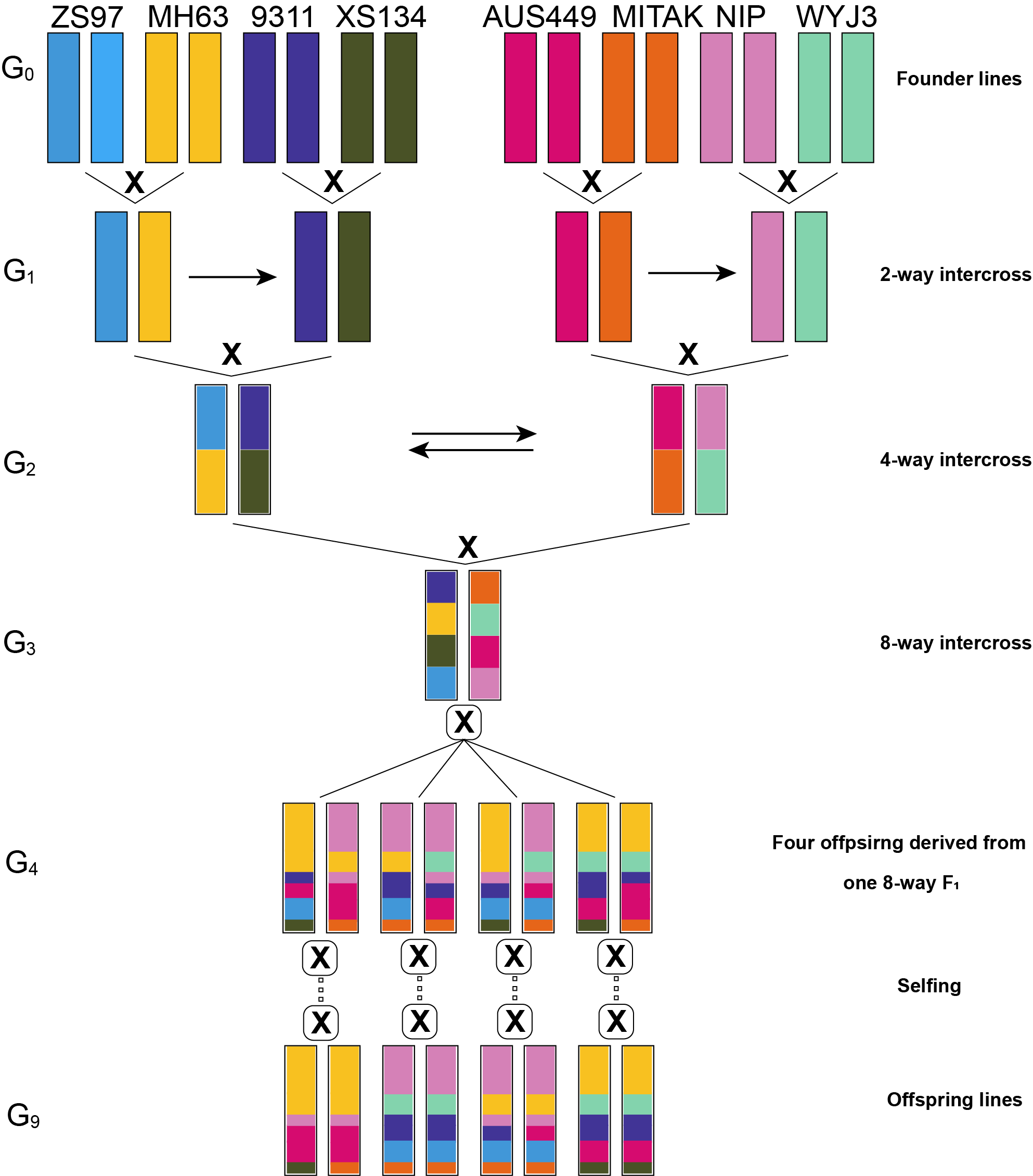


**Supplementary Fig. 22** The construction process of the MAGIC population. Arrows represent the direction of pollen transfer
