# Supplementary material for: Powerful QTL mapping and favorable allele mining in an all-in-one population: a case study of heading date
Source: Natl Sci Rev. 2024 Jun 26;11(8):nwae222. doi: 10.1093/nsr/nwae222 (PMC11360186; doi:10.1093/nsr/nwae222)
Supplement: nwae222_Supplemental_File [file nwae222_supplemental_file.zip › supMethodandMaterials.docx]

**Materials and methods**

**Population design**

By conducting phylogenetic analyses and assessing ancestral component relationships among the 3K germplasms, we identified eight outstanding germplasms with diverse subgroups and geographic representation. These include the progenitors of the highly regarded hybrid Shanyou 63, East Asian type Zhenshan 97 (ZS97) and IRRI type Minghui 63 (MH63). Additionally, the set comprises the widely employed backbone parent restorer line 9311, South Asian circum-Aus type AUS449, and three temperate Geng rice varieties, Nipponbare (NIP), Xiushui 134 (XS134), and Wuyujing 3 (WYJ3), from distinct regions. Last, a tropical Geng rice variety, MITAK, originating from Southeast Asia, completes the selected germplasm collection (Table S18; sup Fig 21).

In the initial phase of hybridization, ZS97 was crossed with MH63, 9311 with XS134, AUS449 with MITAK, and NIP with WYJ3, resulting in the production of four 2-way F_1_ hybrids. In the subsequent phase, ZS97/MH63 and 9311/XS134, AUS449/MITAK and NIP/WYJ3 were crossbred to generate two 4-way F_1_ combinations. In the final phase, the two 4-way F_1_ combinations were hybridized to yield an 8-way F_1_ combination. The authenticity of each generation's hybrids was verified using SSR markers. Subsequently, 291 8-way F_1_ hybrids were self-pollinated to produce 291 F_2_ families, with 10 plants being grown per F_2_ family. From each F_2_ family, four plants were randomly selected to generate RILs through single seed descent. Finally, a total of 1141 inbred lines were obtained in the F_7_ generation, of which 1021 were successfully sampled and sequenced and thus used in further analysis (sup Fig 22).

**Phenotyping**

All 1021 offspring lines and their eight founders were planted at Huazhong Agricultural University on May 17, 2021, and May 19, 2022, in Wuhan. Field trials were carried out following a randomized complete block design. There were two replicates in 2021 and 2022 in Wuhan. Each MAGIC line included 12 individuals per replicate in 2021, and one included 6 individuals in 2022. The 30-day-old seedlings each line was transplanted into one row in one replicate, with a distance of 16.5 cm between plants within a row and 26.4 cm between the rows. The heading date was the number of days from sowing to the appearance of the first panicle. The average value for each inbred line was calculated across all replicates for further analysis. The heritability formula was as below:

$$h2= \frac{Vg}{Vg+ \frac{Vgy}{y}+\frac{Ve}{yr}}$$

In which, Vg is the genetic variance, Vgy is the interaction variance between variety and environment, Ve is the residual, y is the number of years, and r is the number of replicates.

**Whole-genome sequencing**

The leaves of the four parental plants (XS134, AUS449, MITAK, WYJ3) were excised during the vegetative growth stage and rapidly frozen using liquid nitrogen. Subsequently, the frozen leaf samples were transported to Novogene (Beijing, China) for the preparation of PacBio CCS HIFI libraries. The resulting libraries exhibited fragment sizes exceeding 10 kilobases (10K) each. The whole genome was sequenced using the PacBio Sequel II platform. A total of 99.2 GB of CCS HIFI data from 4 founders (~62x coverage for each) was obtained.

Leaves from 1021 offspring families during the vegetative growth stage were collected and stored at a temperature of -20°C. Subsequently, the leaf samples were transported to GENOSEQ (Wuhan, China) for the construction of next-generation sequencing libraries. The libraries were prepared for paired-end whole-genome sequencing using the Illumina HiSeq X sequencing platform. A total of 3.2 TB of sequencing data size from 1021 RILs (~13x coverage for each) was obtained.

**Genome assembly**

Initially, the HIFI reads of the four parents were assembled into an initial skeleton contig using Hifiasm v0.18[1]. To avoid potential misassembly resulting from the low heterozygosity rate of the species, the assembly process incorporated the "-k0" parameter, which prevented duplicate data filtering. Subsequently, HiCanu and FALCON were employed to generate separate contig versions[2, 3]. The contigs obtained from these three assembly approaches were then compared to the gap-free genome MH63, with the Hifiasm results serving as the initial scaffold. Manual error correction was conducted using the HiCanu and FALCON outcomes. As a result, four draft genomes with high integrity were obtained. To fill in the gaps present in the draft genome, the DEGAP pipeline was utilized to align the HIFI reads to the draft genome[4]. Following this, three rounds of polishing were performed on the draft genome. The HIFI reads were compared to the genome using minimap2, and Racon software was used to refine and improve the accuracy of the genome[5, 6]. Ultimately, this process yielded the final polished genome.

Genome integrity was evaluated through the application of two analysis methods: Benchmarking Universal Single-Copy Orthologs (BUSCO) and LTR Assembly Index (LAI)[7, 8]. The BUSCO assessment involved the utilization of the embryophyta_odb10 library, which encompasses a set of 1614 genes known to be universal among embryophytes. To determine the LTR assembly index and identify LTR retrotransposons, we employed three modules: LTR_retriever, LTRharvest, and LTR_FINDER_parallel.

**Genome annotation**

The gene structures of the four genomes were annotated using the MAKER pipeline, considering gene models and annotation evidence[9]. The Augustus model employed was the rice standard model, while the SNAP model was derived from the Rice Population Reference Panel (RPRP)[10]. Various sources of annotation evidence were utilized, including transcripts from PacBio high-quality full-length transcripts, Illumina RNA-Seq transcripts, and the Oryza EST library. Protein data derived from nonredundant genes encoding proteins in Oryza obtained from the RPRP were also considered.

**Collinearity analysis**

To assess the macrocollinearity of the genome sequences, pairwise genome comparisons were performed using minimap2 with the parameter "-asm 5". The resulting Paf alignment file was then utilized to generate a genome-wide collinearity map using NGenomeSyn software[11].

To evaluate the macrocollinearity of genes, MCScanX was employed to identify homologous gene clusters[12]. Homologous clusters containing fewer than 20 genes were filtered out, and a genome-wide gene collinearity map was constructed using NGenomeSyn software.

To evaluate the microcolinearity of the genome genes, the Genetribe pipeline was utilized to identify the homology relationships among the genes of the eight parents[13]. Subsequently, the R package gggenomes (https://github.com/thackl/gggenomes) was employed to visualize the homology results.

**Gene-based pangenome analysis**

The longest transcript from each parent was selected as a representative, and Orthofinder was employed to cluster the genes of each parent[14]. All genes annotated on the eight genomes were clustered together by similarity, including gene absent in Nipponbare. As a result, we obtained a collection of 65236 nonredundant gene sets. The gene set that is present in all eight genomes represents the core gene set, while the gene set present in 2 to 7 genomes represents the dispensable gene set. Additionally, the gene set found in only one particular variety represents the private gene set. To visualize and simulate the pangene maps of both the core and noncore gene sets, PanGP (https://pangp.zhaopage.com/index.html) was utilized. The ComplexUpset (https://github.com/krassowski/complex-upset) package in R was employed to visualize the number of gene sets.

**Variant identification in founders**

The seventh edition of the Nipponbare genome was selected as the reference. The nucmer module of the mummer software was employed to compare the chromosomes of the remaining seven gene components with the reference genome[15]. The comparison was performed with the parameters "--maxmatch -l 50 -c 100". The resulting alignments were filtered using a delta filter to obtain one-to-one linear alignment blocks, with parameters "-m -i 90 -l 100". The output was then converted into the required format for downstream analysis using the show-coords module. SNPs, INDELs, and structural variants within the chromosomes were identified using SyRI with default parameters[16]. Translocations across chromosomes were detected using the combination of minimap2 and SyRI. A custom script was utilized to extract the variations within the homologous regions, generating the final set of variation results. The potential functions of all collinear variants were annotated using SnpEff software[17].

**Variant identification in offspring lines**

The data obtained after splitting were processed using the default parameters of fastp software to eliminate adapter sequences and low-quality sequences[18]. Quality assessment of both the raw and clean data was performed using FastQC (https://www.bioinformatics.babraham.ac.uk/projects/fastqc/). The resulting clean data were aligned to the Nipponbare genome using BWA-MEM, and the alignment files were sorted and compressed using SAMtools[19, 20]. PCR duplicates were removed using the MarkDuplicates module of GATK[21]. Individual genetic variants were identified using the HaplotypeCaller module, and joint genotyping was carried out using the GenotypeGVCFs module.

To obtain high-quality genotypes, we implemented a series of filtering steps. First, for SNPs, we applied quality control criteria such as QD < 2.0, FS > 60, MQ < 40, SOR > 3.0, MQRankSum < -12.5, and ReadPosRankSum < -8.0. Second, SNPs with more than two alleles and those with read depths below half or exceeding twice the average sequencing depth were excluded. Third, SNPs with a missing rate exceeding 20% or a minor allele frequency below 5% were removed. Fourth, SNPs displaying polymorphisms among non-eight parents were excluded. Last, only SNPs present in the collinear region from eight parents were retained. Overall, a total of 2,184,595 SNPs met these filtering criteria.

**Population structure**

LD filtering was conducted on the population genotype using PLINK (https://www.cog-genomics.org/plink/). The entire genome was scanned using a window size of 50 SNPs and a step size of 10 SNPs. Any SNP exhibiting a high correlation coefficient (r2 > 0.2) with other SNPs within the window was excluded. This process yielded a final set of 63748 independent SNPs. Principal component analysis was performed on the filtered SNP set using the "--pca" parameter in PLINK software. The pairwise identity-by-state (IBS) genetic distance between individuals was calculated using the "--distance 1-ibs" parameter in PLINK software. Subsequently, the phylogenetic tree was constructed using the neighbor-joining method in MEGA-X software, and visualization was accomplished using ggtree[22, 23].

**Linkage-disequilibrium analysis**

PLINK software was utilized to compute the linkage disequilibrium (LD) relationship (r2) between pairwise SNPs. The parameters included “--r2 --ld-window-kb 500 --ld-window 999999 --ld-window-r2 0”. The distribution of LD across the genome was assessed by calculating the average value of LD within windows of 500 kb using a step size of 100 kb. Additionally, LD decay was estimated by calculating the average LD value between pairwise SNPs at varying distances using a step size of 1 kb up to a maximum distance of 500 kb.

**Construction of bin map**

To capture the multiallelic advantage of the eight parents, we employed a hidden Markov model to reconstruct the genome-wide bin genotype of the MAGIC population. The fundamental principle involved decoding the sequence of known SNP genotypes and determining the hidden state that most likely corresponded to the arrangement of SNPs, representing the parental genotypes. Considering the specific design of funnel hybridization and six generations of selfing, each site exhibited only eight homozygous parental genotypes and 16 heterozygous parental genotypes. The sum of initial probability for all homozygous parental types was 0.984375, while the sum of initial probability for heterozygous parental types was 0.015625. The transition probability was determined by the physical position of the SNP in the genome, here the transition probability was estimated by recombination rate (1-r)^2^/(8+16r) of the eight-way MAGIC population, where r represents the genetic distance between the two consecutive SNPs, approximately estimated by the physical distance supposing 1 cM covered 250 kb[24]. The emission probability depended on the SNP genotypes of both the founders and offspring lines, as well as the genotyping error rate. For example, if the genotyping error rate was 0.01 and the offspring line had SNP type C while founders A and B had SNP type C, the emission probabilities of AA, BB and AB genotypes are 0.33, while the emission probabilities of other genotypes are 0.0048 and sum to 0.01. Calculations were performed at the chromosome level to determine the final probability of each parental genotype arrangement for each chromosome in each offspring line. The Viterbi algorithm was then employed to backtrack and identify the optimal parental genotype arrangement. Taking chromosomes as units, we calculated the final probabilities of various parental type order for each line, and finally used the Viterbi algorithm to backtrack to the optimal parental type order. Because we only retain polymorphic sites before reconstructing the bin type, if the true recombination breakpoint exists in a non-polymorphic genomic region, the calculated recombination breakpoint is the first mutation point downstream of the true recombination breakpoint. All calculations were conducted using the R package mpmap2 (https://github.com/rohan-shah/mpMap2).

To enhance the accuracy of bin genotypes, we implemented three correction steps as follows: First, for the four families derived from the same 8-way F1 combination, the presence of more than three allele types within a particular genomic region indicated potential errors. In such cases, we traced back to the genome to identify the correct genotype and rectify any erroneous genotypes. Second, we set a minimum threshold for individual recombination fragments at 100 kb. Recombination events smaller than this threshold were uniformly assigned the upstream genotypes to ensure consistency. Third, at the population level, we defined a minimum bin length of 5 kb. Bins shorter than this threshold were filled with the genotypes obtained from the upstream regions. For the case, when multiple founders share the same genotypes over a long region extending up to hundreds of kilobase pairs, the haplotype blocks surrounding the region were used to determine a certain founder contributing to the region of a MAGIC line. That is, the parent shared the same haplotype block as the MAGIC line did is regarded as the contributor of the region because a recombinant bin in a single MAGIC line is frequently more than 1 Mb. By implementing these correction steps, we successfully obtained a total of 24,144 bin types across the entire genome.

**Segregation distortion analysis**

The departure of each bin from the expected Mendelian segregation ratio was evaluated using a chi-square test. The chisq.test function in R performed a chi-square goodness-of-fit test for theoretical proportion of 1/8 from all 8 parents. Using a significance threshold of P = 10^-8^, regions with significant deviations were identified. These regions were further divided into blocks based on strong linkage disequilibrium relationships (D' > 0.6), and the bin with the most pronounced deviation within each block was designated the lead bin. The estimation of linkage disequilibrium parameters for the multi-allele bins was performed using the R package gap (https://cran.r-project.org/web/packages/gap/index.html).

**Recombination hotspot identification**

The genetic linkage map was constructed using GAPL software[25]. The markers within the population were grouped based on their chromosomal information to reduce computational complexity. Within each linkage group, the markers were sorted using the K nearest neighbor algorithm, and their recombination distances were calculated by the Kosambi function. The optimal arrangement order of all markers within each group was determined by summing the adjacent recombination scores.

Using the constructed genetic linkage map, the genome-wide recombination rate of the MAGIC population was computed using the MareyMap website, and a LOESS regression was applied to re-evaluate the recombination rate[26]. The top 5% and bottom 5% of the recombination rate were identified as recombination hotspots and recombination suppression, and regions 5 kb upstream and downstream of the recombination breakpoints were selected. To establish a control, random sequences of the same length and number were selected from the entire genome.

**Genome-wide association analysis**

Genome-wide association analysis was conducted using the FarmCPU model, which is implemented in the R package rMVP[27]. The analysis included the incorporation of the first five principal components as covariates and the use of kinship to control for population structure. Benjamini and Hochberg approach was used to set the threshold value for GWAS. Then *p* = 4.4e-6 as the cut-off for GWAS at genome level. The phenotypic explained variation rate of lead SNP is calculated with previous study[28]. The LDBlockShow software were used to generate local manhattan plot and linkage disequilibrium heatmap[29].

**Linkage analysis**

Genetic mapping analysis was conducted using GAPL software. Additive QTLs were identified using the ICIM (Inclusive Composite Interval Mapping) model. Individuals with missing phenotypes were excluded from the analysis, and a step distance of 0.1 cM and a PIN value of 0.0002 were used. A LOD threshold of 4.0 was determined by permutation test (n = 1000, *P* < .05) at genome level for heading date. The phenotypic explained variation rate of lead SNP is calculated by GAPL.

**Multiomics analysis**

The resequencing data of 529 core germplasm resources were sourced from the PRJNA171289 project, while the transcriptome data of the flag leaf were obtained from project PRJNA858547. For the ZS97 variety, the transcriptome and ATAC-seq data were obtained from project PRJNA705005. Additionally, the methylation and histone modification data of ZS97 were retrieved from the RiceENCODE website.

The genotyping of the 529 core germplasm resources was carried out using the VG pipeline (https://github.com/vgteam/vg). Initially, a graph genome index was constructed using a VCF file containing known genetic variations. Subsequently, the paired-end sequencing data were mapped to the graph genome using Giraffe, and the pack and call modules were utilized to determine the genotype of each variety based on the known genetic variations.

Transcriptome quantification involved mapping the clean reads to the reference genome using HISAT2, followed by transcript assembly and gene quantification using StringTie[30, 31]. In the case of the ZS97 transcriptome, gene expression levels were measured as the mean transcripts per million (TPM) from three biological replicates.

For chromatin accessibility quantification, clean reads were aligned to the reference genome using BWA. Subsequently, PCR duplicates were removed using the sorting functionality of SAMtools and the MarkDuplicates module of GATK. Finally, the bamcoverage module of Deeptools was employed to quantify chromatin accessibility[32].

DNA methylation quantification included mapping clean reads to the reference genome using Bowtie2, removing PCR duplicates with the deduplicate_bismark module of Bismark, and extracting methylation information using the bismark_methylation_extractor module of Bismark[33, 34]. Methylation files with a sequencing depth greater than or equal to 3 for CG/CHG/CHH cytosine positions were converted into bedgraph format for quantification.

Histone modification quantification involved mapping clean reads to the reference genome using Bowtie2, retaining reads with a quality score greater than 30 using SAMtools, removing PCR duplicates using the MarkDuplicates module of GATK, and quantifying the histone modifications using the bamcoverage module of Deeptools.

**Reference**

1. Cheng HY, Concepcion GT, Feng XW *et al.* Haplotype-resolved de novo assembly using phased assembly graphs with hifiasm. *Nat Methods*. 2021; **18**(2): 170-+. doi: 10.1038/s41592-020-01056-5

2. Nurk S, Walenz BP, Rhie A *et al.* HiCanu: accurate assembly of segmental duplications, satellites, and allelic variants from high-fidelity long reads. *Genome Res*. 2020; **30**(9): 1291-1305. doi: 10.1101/gr.263566.120

3. Chin CS, Peluso P, Sedlazeck FJ *et al.* Phased diploid genome assembly with single-molecule real-time sequencing. *Nat Methods*. 2016; **13**(12): 1050-+. doi: 10.1038/Nmeth.4035

4. Huang Y, Wang Z, Schmidt MA *et al.* DEGAP: Dynamic Elongation of a Genome Assembly Path. *bioRxiv*. 2023: 2023.2004. 2025.538224.

5. Vaser R, Sovic I, Nagarajan N *et al.* Fast and accurate de novo genome assembly from long uncorrected reads. *Genome Res*. 2017; **27**(5): 737-746. doi: 10.1101/gr.214270.116

6. Li H. Minimap2: pairwise alignment for nucleotide sequences. *Bioinformatics*. 2018; **34**(18): 3094-3100. doi: 10.1093/bioinformatics/bty191

7. Simao FA, Waterhouse RM, Ioannidis P *et al.* BUSCO: assessing genome assembly and annotation completeness with single-copy orthologs. *Bioinformatics*. 2015; **31**(19): 3210-3212. doi: 10.1093/bioinformatics/btv351

8. Ou SJ, Chen JF, Jiang N. Assessing genome assembly quality using the LTR Assembly Index (LAI). *Nucleic Acids Res*. 2018; **46**(21). doi: 10.1093/nar/gky730

9. Cantarel BL, Korf I, Robb SMC *et al.* MAKER: An easy-to-use annotation pipeline designed for emerging model organism genomes. *Genome Res*. 2008; **18**(1): 188-196. doi: 10.1101/gr.6743907

10. Zhou Y, Chebotarov D, Kudrna D *et al.* A platinum standard pan-genome resource that represents the population structure of Asian rice. *Sci Data*. 2020; **7**(1). doi: ARTN 113

10.1038/s41597-020-0438-2

11. He WM, Yang J, Jing Y *et al.* NGenomeSyn: an easy-to-use and flexible tool for publication-ready visualization of syntenic relationships across multiple genomes. *Bioinformatics*. 2023; **39**(3). doi: ARTN btad121

10.1093/bioinformatics/btad121

12. Wang YP, Tang HB, DeBarry JD *et al.* MCScanX: a toolkit for detection and evolutionary analysis of gene synteny and collinearity. *Nucleic Acids Res*. 2012; **40**(7). doi: ARTN e49

10.1093/nar/gkr1293

13. Chen YM, Song WJ, Xie XM *et al.* A Collinearity-Incorporating Homology Inference Strategy for Connecting Emerging Assemblies in the Triticeae Tribe as a Pilot Practice in the Plant Pangenomic Era. *Mol Plant*. 2020; **13**(12): 1694-1708. doi: 10.1016/j.molp.2020.09.019

14. Emms DM, Kelly S. OrthoFinder: phylogenetic orthology inference for comparative genomics. *Genome Biol*. 2019; **20**(1). doi: ARTN 238

10.1186/s13059-019-1832-y

15. Marcais G, Delcher AL, Phillippy AM *et al.* MUMmer4: A fast and versatile genome alignment system. *Plos Comput Biol*. 2018; **14**(1). doi: ARTN e1005944

10.1371/journal.pcbi.1005944

16. Goel M, Sun HQ, Jiao WB *et al.* SyRI: finding genomic rearrangements and local sequence differences from whole-genome assemblies. *Genome Biol*. 2019; **20**(1). doi: ARTN 277

10.1186/s13059-019-1911-0

17. Cingolani P, Platts A, Wang LL *et al.* A program for annotating and predicting the effects of single nucleotide polymorphisms, SnpEff: SNPs in the genome of Drosophila melanogaster strain w(1118); iso-2; iso-3. *Fly*. 2012; **6**(2): 80-92. doi: 10.4161/fly.19695

18. Chen SF, Zhou YQ, Chen YR *et al.* fastp: an ultra-fast all-in-one FASTQ preprocessor. *Bioinformatics*. 2018; **34**(17): 884-890. doi: 10.1093/bioinformatics/bty560

19. Li H. Aligning sequence reads, clone sequences and assembly contigs with BWA-MEM. *arXiv*. 2013.

20. Danecek P, Bonfield JK, Liddle J *et al.* Twelve years of SAMtools and BCFtools. *Gigascience*. 2021; **10**(2). doi: 10.1093/gigascience/giab008

21. McKenna A, Hanna M, Banks E *et al.* The Genome Analysis Toolkit: A MapReduce framework for analyzing next-generation DNA sequencing data. *Genome Res*. 2010; **20**(9): 1297-1303. doi: 10.1101/gr.107524.110

22. Kumar S, Stecher G, Li M *et al.* MEGA X: Molecular Evolutionary Genetics Analysis across Computing Platforms. *Mol Biol Evol*. 2018; **35**(6): 1547-1549. doi: 10.1093/molbev/msy096

23. Yu GC, Smith DK, Zhu HC *et al.* GGTREE: an R package for visualization and annotation of phylogenetic trees with their covariates and other associated data. *Methods Ecol Evol*. 2017; **8**(1): 28-36. doi: 10.1111/2041-210x.12628

24. Broman KW. The Genomes of Recombinant Inbred Lines. *Genetics*. 2005; **169**(2): 1133-1146. doi: 10.1534/genetics.104.035212

25. Zhang LY, Meng L, Wang JK. Linkage analysis and integrated software GAPL for pure-line populations derived from four-way and eight-way crosses. *Crop J*. 2019; **7**(3): 283-293. doi: 10.1016/j.cj.2018.10.006

26. Siberchicot A, Bessy A, Gueguen L *et al.* MareyMap Online: A User-Friendly Web Application and Database Service for Estimating Recombination Rates Using Physical and Genetic Maps. *Genome Biol Evol*. 2017; **9**(10): 2506-2509. doi: 10.1093/gbe/evx178

27. Yin LL, Zhang HH, Tang ZS *et al.* rMVP: A Memory-efficient, Visualization-enhanced, and Parallel-accelerated Tool for Genome-wide Association Study. *Genom Proteom Bioinf*. 2021; **19**(4): 619-628. doi: 10.1016/j.gpb.2020.10.007

28. Sun L, Li X, Fu Y *et al.* GS 6, a member of the GRAS gene family, negatively regulates grain size in rice. *Journal of integrative plant biology*. 2013; **55**(10): 938-949.

29. Dong S-S, He W-M, Ji J-J *et al.* LDBlockShow: a fast and convenient tool for visualizing linkage disequilibrium and haplotype blocks based on variant call format files. *Briefings in Bioinformatics*. 2020; **22**(4). doi: 10.1093/bib/bbaa227

30. Kim D, Landmead B, Salzberg SL. HISAT: a fast spliced aligner with low memory requirements. *Nat Methods*. 2015; **12**(4): 357-U121. doi: 10.1038/Nmeth.3317

31. Pertea M, Pertea GM, Antonescu CM *et al.* StringTie enables improved reconstruction of a transcriptome from RNA-seq reads. *Nature Biotechnol*. 2015; **33**(3): 290-+. doi: 10.1038/nbt.3122

32. Ramirez F, Ryan DP, Gruning B *et al.* deepTools2: a next generation web server for deep-sequencing data analysis. *Nucleic Acids Res*. 2016; **44**(W1): W160-W165. doi: 10.1093/nar/gkw257

33. Langmead B, Salzberg SL. Fast gapped-read alignment with Bowtie 2. *Nat Methods*. 2012; **9**(4): 357-U354. doi: 10.1038/Nmeth.1923

34. Krueger F, Andrews SR. Bismark: a flexible aligner and methylation caller for Bisulfite-Seq applications. *Bioinformatics*. 2011; **27**(11): 1571-1572. doi: 10.1093/bioinformatics/btr167
